# Solid State Reactions Involving Oxides of Trivalent Cations

**DOI:** 10.6028/jres.065A.037

**Published:** 1961-08-01

**Authors:** S. J. Schneider, R. S. Roth, J. L. Waring

## Abstract

Selected mixtures in 69 binary systems involving Al_2_O_3_, Ga_2_O_3_, Cr_2_O_3_, Fe_2_O_3_, Sc_2_O_3_, In_2_O_3_, Y_2_O_3_, and the rare earth oxides were studied by X-ray diffraction techniques after heat treatment at various temperatures. A plot of the radii of the A^+3^ cations versus the radii of B^+3^ cations shows the regions of stability for the different structure types found for the double oxides of the trivalent cations. The following structure types were encountered: A, B, and C-type rare earth oxide; corundum, beta gallia; kappa alumina; garnet; perovskite; and several types which could not be definitely related to known structures. The majority of A^+3^B^+3^O_3_ compounds have the perovskite structure. Several phases, including (1−*x*)Fe_2_O_3_·*x*Al_2_O_3 ss_ and (1−*x*)Fe_2_O_3_·Ga_2_O_3 ss_, appear to have structures similar to kappa alumina. Solid solution definitely occurs in many of the garnet type compounds which contain gallia. Based on the data collected in this survey, the subsolidus phase equilibria relationships of 79 binary systems were drawn.

## 1. Introduction

In the field of phase equilibria research it is often beneficial to first survey a series of related systems before commencing on a detailed analysis of specific systems. A survey was recently conducted by the authors [1]^1^ on the various solid state reactions that occur in mixtures of the trivalent rare earth oxides. It was found in the work that ionic size was the primary controlling factor in determining the various subsolidus phase relationships. This study has since been extended to include the oxides of the smaller trivalent cations, In^+3^, Sc^+3^, Fe^+3^, Cr^+3^, Ga^+3^, and Al^+3^. These cations, together with the lanthanide series comprise almost the entire group of ions which are commonly trivalent.

To date, only a limited number of binary oxide systems involving only trivalent cations have been completely studied. With the exception of the previously mentioned paper by Schneider and Roth [1], most of the research has been concerned with studies of A^+3^B^+3^O_3_ and to a lesser extent A_3_^+3^B_5_^+3^O_12_ type compounds. The A^+3^B^+3^O_3_ and A_3_^+3^B_5_^+3^O_12_ compounds have the perovskite and garnet structures respectively. It is noteworth that the oxides of the trivalent cations, A_2_^+3^O_3_, may be considered in a general way as A^+3^B^+3^O_3_ (A^+3^A^+3^O_3_) type compounds. None of these A_2_^+3^O_3_ oxides, however, are known to have a perovskite structure. Goldschmidt and his coworkers [2] were perhaps the first to investigate A^+3^B+^3^O_3_ compounds in detail. Many other investigators, including Keith and Roy [3], Roth [4], and Geller and his coworkers [5, 6, 7] have substantially contributed to the data available on this formula-type compound.

The purpose of the present investigation was to survey the various structure types that occur under equilibrium conditions for different binary mixtures of the oxides of the trivalent cations and to establish the subsolidus phase equilibria relationships for various systems. Special emphasis was given to a classification of the structure types found for equimolar mixtures according to the ionic radii of the constituent cations.

## 2. Sample Preparation and Test Methods

With the exception of Cr_2_O_3_ and Fe_2_O_3_ which were reagent grade, the materials used in this investigation had a purity of about 99.9 percent. Specimens were prepared from either 0.5 or 1.0 gram batches of various binary combinations of different oxides. Calculated amounts of each end member, corrected for ignition loss, were weighed to the nearest milligram. Each batch was mixed, formed into a ⅜ in.-diam pellet by pressing at 10,000 lb/in.^2^ and fired at some relatively low temperature (at least 800 °C) for varying lengths of time. Most of the specimens were then ground, remixed, again pressed into pellets and fired at successively higher temperatures until equilibrium was obtained.

All specimens containing In_2_O_3_ or Cr_2_O_3_, were ground, mixed, and then sealed in platinum tubes for the higher temperature heat treatments. The duration and temperature of each heat treatment generally varied with the particular system under consideration. In general, the specimens were slow cooled at approximately 4 °C/min. However, a few of the mixtures were quenched from elevated temperatures.

All heatings were performed in an air atmosphere using a conventional muffle furnace for the low temperature heats and a program-controlled tube furnace or a manually operated quench furnace for the heat treatment between 1000 and 1650 °C. An induction furnace, having as the susceptor a small iridium crucible, was used for heat treatments above 1650 °C. Temperatures were controlled to at least ±10 °C.

Equilibrium was considered to have been attained when the X-ray patterns of a specimen showed no change with successive heat treatment of the specimen or when the X-ray powder data was consistent with the results predicted from a previous set of experiments. All specimens were examined at room temperature by X-ray diffraction with a Geiger-counter diffractometer employing nickel-filtered copper radiation.

## 3. Results

The data obtained in this investigation are given in table 1. The table lists six groups of binary systems, each having either Al_2_O_3_, Ga_2_O_3_, Cr_2_O_3_, Fe_2_O_3_, Sc_2_O_3_, or In_2_O_3_ as one component. Each of these groups in turn is arranged according to decreasing cation size of the second component. Selected literature references are included for compositions not studied experimentally in the present work. No attempt was made to incorporate into the table any data pertaining solely to mixtures involving only oxides of the trivalent rare earth cations. These data were reported in a recent publication by Schneider and Roth [1]. The table was designed primarily to present sufficient data to estimate the subsolidus phase relationships of a majority of the listed binary systems.

**Figure 1:**
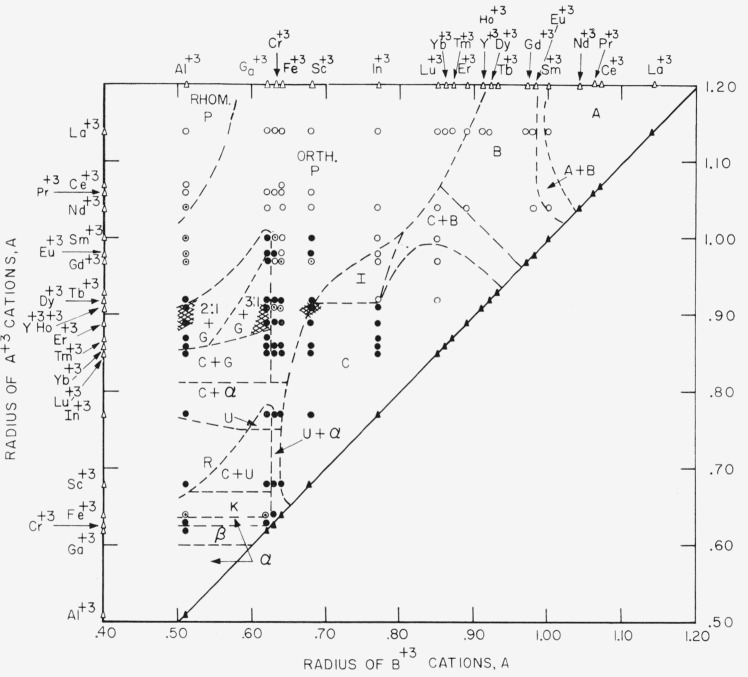
Classification of structure types for equimolar mixtures of trivalent cations. Δ—radii of cations ▲—pure oxide ●—compositions studied in present work ○—data taken from literature A—A-type rare earth oxide B—B-type rare earth oxide C—C-type rare earth oxide G—garnet I—unknown type *α*—corundum *β*—beta gallia K—kappa alumina U—unknown type similar to kappa alumina P—perovskite R—unknown type 2:1—unknown type 3:1—unknown type

Figure 1 gives a classification of the various structure types found for equimolar mixtures of the oxides of the trivalent cations. The coordinates of the figure are the radii of the A^+3^ and B^+3^ cations. For convenience the larger cation in any mixture is taken as the A^+3^ cation (ordinate) and the smaller as B^+3^ (abscissa). The radii of the different cations are indicated on the figure by open triangles.^2^ The solid triangles on the diagonal line represent the individual oxides. Each circle represents an equimolar composition containing either one or two phases which have the indicated structures at room temperature. In most instances, these same types also exist stably at elevated temperatures. The one known exception to this is the listed structure of the 1:1 mixture of Fe_2_O_3_ and Al_2_O_3_ which is metastable at room temperature [10]. The diagram does not indicate any reversible phase transformations or decompositions that occur at elevated temperatures. It should be emphasized that the boundaries outlining each field were arbitrarily drawn. They do not indicate the division of different structure types for solid solutions which may exist between adjacent equimolar mixtures.

The occurrence of metastable phases was prevalent in a number of the double oxides which are near the boundary lines of figure 1. It was extremely difficult at times to establish the equilibrium phases. For this reason certain areas in the diagram are shaded to indicate that the position of certain portions of the boundary lines are somewhat in doubt. Several of these borderline systems are now being investigated in detail by the authors order that the equilibrium phases can be definitely ascertained.

The majority of A^+3^B^+3^O_3_ type compounds formed from double oxides of the trivalent cations are those having the perovskite structure. This field of perovskite types encompasses the largest single phase area of the diagram. The other single phase areas generally represent solid solutions and not true compounds. The two-phase areas, of course, contain compounds (3:1, 2:1, and 3:5) but not of the A^+3^B^+3^O_3_ type.

Figure 1 not only designates the structure types for equimolar mixtures but also, with two exceptions, is applicable for all molar ratios of binary combinations of oxides of the listed cations. The two exceptions are the beta alumina (La_2_O_3_-Al_2_O_3_ and La_2_O_3_-Fe_2_O_3_ systems) and the spinel (Fe_2_O_3_-R_2_O_3_ systems) structures. The spinel structure, of course, occurs only when FeO is present as a third component. The various structure types are discussed in succeeding sections.

## 4. Discussion

### 4.1. A, B, C, Beta Gallia, and Corundum Structure Types

The structure type of the stable forms of the oxides of the trivalent cations (fig. 1, solid triangles) can be generally grouped in the following manner according to the ionic radius of the constituent cations: 1.14 A to 1.04 A-hexagonal A-type rare earth oxide structure; 1.00 A to 0.97 A-monoclinic B-type rare earth oxide structure; 0.93 A to 0.68 A-cubic C-type rare earth oxide structure; 0.64 A to 0.63A-rhombohedral corundum structure; 0.62 A-monoclinic beta gallia structure; and 0.51 A-rhombohedral corundum structure. In the above listing the structure types are seemingly out of order with respect to radii in that the beta gallia type is intermediate between two corundum types. This inconsistency emphasizes that other factors besides radii must be considered in generalizations such as given above.

Generally the effects of partial covalent bonding in essentially ionic type materials are neglected. Mooser and Pearson [11] related the structures of certain simple compounds to average quantum numbers and electronegativity values. From their work and others [11, 12] it is apparent that the covalent character (directional properties of the bonds) of a compound is directly related to the difference in the electronegativities of the cation and anion. Generally the greater the difference, the less the covalent type bonding. Using the electronegativity values given by Gordy and Thomas [13] to calculate relative covalent character, the aforementioned grouping of structure types can be rearranged according to increasing covalent character: A, B, or C types (Ln_2_O_3_)^3^ <C-type (Sc_2_O_3_)<C-type (In_2_O_3_)<beta gallia type (Ga_2_O_3_)<corundum type (Al_2_O_3_)<corundum type (Cr_2_O_3_)<corundum type (Fe_2_O_3_). This method of arrangement, although on a very relative scale, does group like structure types together. It would be increasingly more difficult to apply this type of classification to compounds containing ions of different valence as well as those containing multiple ions of the same valence.

A number of the trivalent oxides have metastable polymorphs which have structures different from the stable modifications. A B-type structure has been reported for Nd_2_O_3_ [14], while Sm_2_O_3_, Eu_2_O_3_, and Gd_2_O_3_ are known to form the C-type [9]. Gallia (Ga_2_O_3_) and Al_2_O_3_ are similar in many respects in that they both have polymorphs of the same structure type. Gamma Al_2_O_3_ and gamma Ga_2_O_3_ are isostructural, as are alpha Al_2_O_3_ and alpha Ga_2_O_3_ [15]. This is also true for epsilon Ga_2_O_3_ and kappa Al_2_O_3_ and for beta Ga_2_O_3_ and theta Al_2_O_3_ [15]. A metastable polymorph of a pure oxide may appear as a stable phase in solid solutions. Examples of this occur in solid solutions between the oxides of the trivalent rare earth ions. For instance, the B-type structure in solid solutions is stable over a far greater range of average radii values than the pure oxides [1].

### 4.2. Perovskite Structure Type

The various combinations of double oxides that form 1:1 compounds which have the perovskite structure are indicated in figure 1. Each of these compounds has modifications which are distorted from the ideal cubic structure assuming either rhombohedral or orthorhombic symmetry at room temperature. At elevated temperatures other symmetries may occur. It has been suggested that the order of transformation with temperature is probably orthorhombic to rhombohedral to cubic [7].

Goldschmidt and coworkers [2] derived a tolerance factor (*t*) for the perovskite structure which is given by the following formula:

$$t=\frac{R_{A}+R_{o}}{\sqrt{2}\left(R_{B}+R_{o}\right)}$$

where

*t*=tolerance factor
*R_A_*=radius of larger cation
*R_B_*=radius of smaller cation
*R_o_*=radius of oxygen (1.40 A).

As *t* approaches unity, the tendency for the formation of a perovskite structure becomes greater. The lower limit or minimum value of *t* for a given series can only be determined experimentally. For the Ln_2_O_3_ Ga_2_O_3_ and Ln_2_O_3_·Al_2_O_3_ series of perovskite type compounds, the minimum values of *t* were found to be 0.85 and 0.84 respectively. In comparison, the lower limit of *t* for the other perovskite type series, La_2_O_3_·Ln_2_O_3_ [1], Ln_2_O_3_·In_2_O_3_, Ln_2_O_3_·Sc_2_O_3_, Ln_2_O_3_·Fe_2_O_3_ and Ln_2_O_3_·Cr_2_O_3_, are all equal to about 0.78.

**Figure 2:**
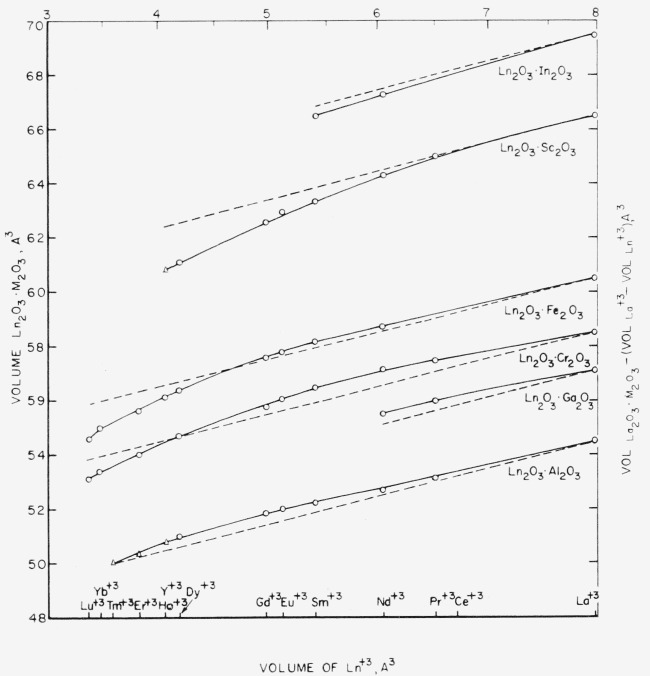
Relationship between volumes of one formula weight of Ln_2_O_3_·M_2_O_3_ perovskites and volumes of cations in 12-fold coordination. Solid Curve—Determined experimentally Dashed Curve—Predicted from lanthanide contraction, $\left[V_{{\text{La}}_{2}O_{3}\cdot M_{2}O_{3}}-\left(V_{\text{La}}-V_{\text{Ln}}\right)\right]$ ⊙—Stable compound Δ—Possibly metastable compound

Dalziel [16], considering only the Fe_2_O_3_, Ga_2_O_3_, and Al_2_O_3_ perovskite series, attempted to explain the differences in minimum *t* values on the basis of partial covalent character of the non rare earth cation-oxygen bond. To test the relative covalent character of the different series, Dalziel presented a graph similar to that given in figure 2. Expanding Dalziel’s graph to include all appropriate data in table 1, figure 2 shows the relationship between the volumes of the Ln^+3^ cations in 12-fold coordination and the volumes of one formula weight of Ln_2_O_3_·M_2_O_3_ perovskite type compounds, as both determined experimentally (solid lines) and as predicted from the lanthanide contraction (dashed lines). For a given series, the volumes should decrease in a regular manner with the lanthanide contraction. The decrease, however, will be modified somewhat from that predicted, due to: (1) the deviation from close packing caused by increased distortion of the lattice and (2) the influence of covalent character of the cation-oxygen bonds [16]. The former would result in larger volumes than those predicted while the latter would produce an opposite effect.

In general, it can be concluded from figure 2 that for a given series, the covalent character significantly increases as the size of the Ln^+3^ cation decreases. It is difficult to compare the different series with regard to which group is more covalent because of masking effects of the various factors. It does appear, however, that the effect of partial covalent bonding is less pronounced in the Al_2_O_3_ and perhaps the Ga_2_O_3_ series than in the other groups. This would account for the larger minimum tolerance factors of the Al_2_O_3_ and Ga_2_O_3_ series.

### 4.3. Garnet Structure Type

The garnet structure occurs at the ideal 3:5 molar ratio in a number of binary systems involving oxides of the trivalent cations. Specifically, these include systems containing either Fe_2_O_3_, Ga_2_O_3_, or Al_2_O_3_ as one end member and a rare earth oxide (or Y_2_O_3_) as the other. The chemical formula of a garnet type compound can be written as [A_3_^+3^][B_2_^+3^][C_3_^+3^]O_12_, where [A^+3^], [B^+3^], and [C^+3^] indicate cations which occur in 8-fold, 6-fold, and 4-fold coordination, respectively [17]. In binary systems the rare earth cations or Y^+3^ can be usually thought of as occupying the [A^+3^] sites with the smaller cations, Fe^+3^, Ga^+3^, or Al^+3^ filling the [B^+3^] and [C^+3^] positions.

Compounds having the garnet structure do not occur in binary systems containing Cr_2_O_3_. This agrees with the observation [17] that Cr^+3^ prefers only octahedral type of coordination ([B^+3^] sites) in the garnet structure. Apparently the Cr^+3^ cations will never appreciably occupy tetrahedral sites in the garnet structure, even when it is the most likely cation to be tetrahedrally coordinated.

**Figure 3:**
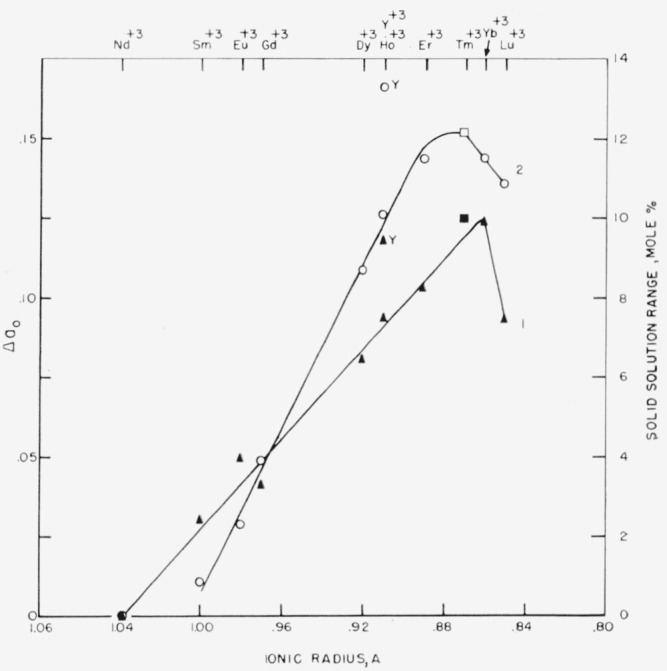
Relationship between ionic radii and both compositional range of solid solution and corresponding parameter change in several gallia garnets at 1500 °C. Curve 1—Solid solution range Curve 2—Change in unit cell dimension Δa_0_=a_0_ 1:1−a_0_ 3:5 □= estimated

Solid solution of the garnet type compounds which occurs in binary systems containing Ga_2_O_3_ has been generally overlooked because of the simultaneous report of solid solution between the perovskite and garnet structures in the Y_2_O_3_-Al_2_O_3_ system [3]. Solid solution definitely occurs in many binary gallia garnets. Figure 3 shows plots of the radii of the rare earth cations against both the compositional range of solid solution of the various garnet compounds (no. 1) and the corresponding change in unit cell dimensions (no. 2). In these garnet solid solutions, the rare earth cation apparently subsitutes for Ga^+3^ in the octahedral ([B^+3^]) positions.^4^

The amount of solid solution as well as the amount of change in unit cell dimensions increases to a maximum at about Tm^+3^ as the size of the constituent rare earth cation decreases. The reason for this behavior is unknown. In addition, the values determined for the garnet solid solution in the Y_2_O_3_-Ga_2_O_3_ system were excessively larger than expected and do not fit the general curves of figure 3.

It is interesting to observe that solid solution of the garnet type compound for the gallia series occurs only in binary systems in which a perovskite-type compound does not exist as a stable phase. On this premise it was considered likely and experimentally verified that solid solution does occur in the smaller alumina garnets, 3Yb_2_O_3_·5Al_2_O_3_ and 3Lu_2_O_3_·5Al_2_O_3_. Although not determined exactly, the extent of garnet solid solution is fairly small, probably about two mole percent. Substitutional type solid solution of the Fe_2_O_3_ garnets probably does not occur. However, as illustrated by the Y_2_O_3_-Fe_2_O_3_ system [18], partial reduction of Fe_2_O_3_ in these garnets may produce solid solution to a limited extent.

### 4.4. Kappa Alumina Structure Type

Considerable confusion exists in the literature with regard to the various low temperature, metastable polymorphs of Al_2_O_3_ and Ga_2_O_3_. These polytypes are ill-defined primarily because of the inability to obtain clear, interpretable X-ray diffraction data. Of particular interest in the present investigation are the kappa alumina and epsilon gallia polymorphs and their characteristic structures. Roy et al. [15] have clearly demonstrated through a series of solid solution studies that kappa alumina and epsilon gallia in reality have the same structure. The alumina polymorph having the kappa alumina structure has been reported [19] to be orthorhombic with *a*=8.49 A, *b* = 12.73 A, and *c*=13.39 A. The reported *d*-spacings were not given with sufficient accuracy to verify the cell dimensions.

Richardson et al. [20] described the phase which occurs at the equimolar mixture of Fe_2_O_3_ and Al_2_O_3_ as having a structure similar to that of kappa alumina. The X-ray pattern for the 50Fe_2_O_3_: 50Al_2_O_3_ phase was indexed by Richardson et al. [20] on the basis of an orthorhombic cell with *a*=7.03 A, *b*=6.33 A, and *c*=7.41 A.^5^ However, the calculated and observed *d*-spacings do not appear to be in close enough agreement to justify the reported indexing. In the present investigation three Fe_2_O_3_-Al_2_O_3_ mixtures, 47:53, 50:50, and 53:47 were prepared. Each specimen contained the same single phase as that reported by Richardson et al. [20]. The X-ray pattern of the 53Fe_2_O_3_:47Al_2_O_3_ specimen was successfully indexed on the basis of an orthorhombic cell with *a*=8.59 A, *b*=9.23 A, and *c*=4.98 A as given in table 2. The indexing was accomplished only after comparison with the X-ray pattern of the 50Fe_2_O_3_:50Ga_2_O_3_ specimen, a phase described by Wood [21]. The orthorhombic phases which occur in the Fe_2_O_3_-Al_2_O_3_ and Fe_2_O_3_-Ga_2_O_3_ systems are apparently isostructural and represent solid solutions rather than compounds. The similarity in structures is important because of the reported magnetic and piezoelectric properties of the (1−*x*)Fe_2_O_3_·*x*Ga_2_O_3_ *_ss_* phase. These properties in (1−*x*)Fe_2_O_3_·*x*Al_2_O_3_ *_ss_* will be reported on in a future publication. Muan and Somiya [10] reported the complete phase relations for the Fe_2_O_3_-Al_2_O_3_ system and showed that the orthorhombic phase has both a minimum and maximum decomposition temperature.

There is not yet sufficient evidence to classify the orthorhombic phases of the Fe_2_O_3_-Al_2_O_3_ and Fe_2_O_3_-Ga_2_O_3_ systems as having a kappa alumina structure although there is a definite similarity. The X-ray patterns given in the literature for the kappa alumina and epsilon gallia polymorphs could not be indexed on the same basis as that given for 53Fe_2_O_3_:47Al_2_O_3_ in table 2. The failure to index these patterns may be due to the inaccurate X-ray data available rather than dissimilar structures.

**Figure 4:**
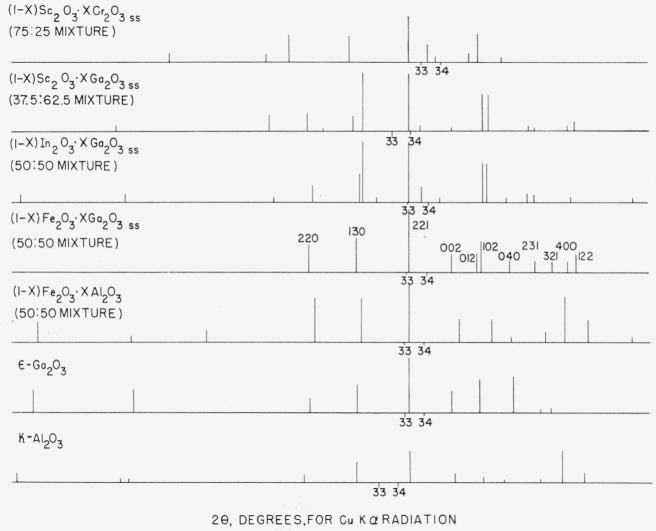
Diagrammatic X-ray powder diffraction patterns for kappa alumina [25], epsilon gallia [15], 50Fe_2_O_3_:50Al_2_O_3_ 50Fe_2_O_3_:50Ga_2_O_3_, 50In_2_O_3_:50Ga_2_O_3_, 37.5Sc_2_O_3_:62.5Ga_2_O_3_ and 75Sc_2_O_3_:25Cr_2_O_3_. For the kappa alumina pattern, d-values apparently due to extraneous phases were deleted, as was done by Roy et al. [15].

Figure 4, as well as table 3, presents X-ray powder data for all the phases encountered in this investigation which may have structures similar to kappa alumina. It is apparent, from figure 4, that the patterns for kappa alumina, epsilon gallia, 50Fe_2_O_3_: 50Al_2_O_3_ and 50Fe_2_O_3_:50Ga_2_O_3_ are related. Each of the X-ray patterns of the other phases, 50In_2_O_3_:50Ga_2_O_3_, 37.5Sc_2_O_3_:62.5Ga_2_O_3_, and 75Sc_2_O_3_:25Cr_2_O_3_, could not be indexed although they, too, appear similar to the pattern of kappa alumina. It would seem, strictly by the comparison of X-ray patterns, that In_2_O_3_-Ga_2_O_3_ and Sc_2_O_3_-Ga_2_O_3_ phases are isostructural with each other, but not necessarily with kappa alumina. The phase most dissimilar with kappa alumina in this entire group is that of 75Sc_2_O_3_:25Cr_2_O_3_.

### 4.5. Other Structure Types

Keith and Roy [3] reported that an unknown phase occurs in a melted 50:50 mixture of In_2_O_3_ and Al_2_O_3_. They designated this phase as a high form of In_2_O_3_:Al_2_O_3_ and listed several of its X-ray reflections. In an effort to obtain this phase, the experiment of Keith and Roy was repeated. The melted specimen of 50:50 In_2_O_3_-Al_2_O_3_ contained two phases, In_2_O_3_ and apparently the same phase as reported by Keith and Roy. Other experiments with the 50In_2_O_3_:50Al_2_O_3_ mixture indicated that the unknown phase is probably metastable in the In_2_O_3_-Al_2_O_3_ system and occurs only on quenching the melt.

In the Sc_2_O_3_-Al_2_O_3_ system a stable phase occurs which, according to X-ray powder data, appears to be isostructural with the metastable phase of the In_2_O_3_-Al_2_O_3_ system. This phase occurs over a region of Sc_2_O_3_-Al_2_O_3_ compositions and represents a solid solution and not a true compound. The X-ray pattern of the 50Sc_2_O_3_:50Al_2_O_3_ mixture is given in table 4. The pattern was indexed on the basis of a rhombohedral cell by comparison with the pattern of 2PbO·Nb_2_O_5_, a rhombohedral distortion of the pyrochlore structure. The X-ray pattern for the Sc_2_O_3_-Al_2_O_3_ phase was diffuse regardless of heat treatment of the specimen, and therefore the agreement between observed and calculated values, given in table 4, is only fair for the less intense reflections. Single crystal data is needed to ascertain the correct structure type. Superstructure peaks, necessary to differentiate a body centered C-type structure or a face centered pyrochlore structure from the fluorite or Sb_2_O_3_-type structures, could not be found in the X-ray pattern. The fluorite structure would require that all the oxygen vacancies be disordered. For the Sc_2_O_3_-Al_2_O_3_ phase to have a C-type or a Sb_2_O_3_-type structure a complete ordering of the vacant oxygen sites would be required while the pyrochlore structure would necessitate only partial ordering.

A number of different phases encountered in this investigation have not been identified or even related with a specific known structure type. These phases, apparently all compounds, exist in various systems at either the 3:1, 2:1 or 1:1 molar compositions.

The 3:1 compounds occur exclusively in galliate systems; specifically, Ga_2_O_3_ with either Sm_2_O_3_, Eu_2_O_3_, Gd_2_O_3_, Dy_2_O_3_, Ho_2_O_3_, Y_2_O_3_, or Er_2_O_3_. These compounds, all apparently isostructural, have not been previously reported. The X-ray data of 3Gd_2_O_3_·Ga_2_O_3_, which is given in table 5, is typical of all the patterns of these isostructural 3:1 compounds. The only difference between the various patterns is the appropriate shift in the *d*-spacings of the X-ray reflections due to cation size differences. It is noteworthy that the 3:1 compound does not occur in systems in which a perovskite type compound forms as a stable phase.

A series of apparently isostructural 2:1 compounds exist in both aluminate and galliate systems. The first 2:1 compound of this type studied extensively was the 2Y_2_O_3_-Al_2_O_3_ phase [22]. In binary aluminate systems, Gd^+3^, Dy^+3^, Ho^+3^, Er^+3^, Tm^+3^, and Yb^+3^ can be substituted for Y^+3^. In galliate systems, however, only the oxides of the larger cations La^+3^, Nd^+3^, Sm^+3^, and Eu^+3^ form 2:1 compounds with Ga_2_O_3_. Evidently this structure type is dependent on radius ratios and will only occur within specific ranges of cation radii values. An example of this occurs in the Yb_2_O_3_-Al_2_O_3_ and Lu_2_O_3_-Al_2_O_3_ systems. The 2:1 compound forms in the Yb_2_O_3_-Al_2_O_3_ system but not in the Lu_2_O_3_-Al_2_O_3_ system, even though the radius of Lu^+3^ is only 0.01 A smaller than that of Yb^+3^. However, the X-ray pattern of the 3:5 mixture in the Lu_2_O_3_-Al_2_O_3_ system showed, in addition to the garnet peaks, a few minor reflections which may represent a 2:1 phase. At present, it would appear that the occurrence of a 2:1 compound in the Lu_2_O_3_-Al_2_O_3_ system is strictly a metastable phenomenon.

Table 6 compares the X-ray pattern for 2Y_2_O_3_·Al_2_O_3_ obtained in this investigation with that reported by Warshaw and Roy [22]. They described this 2:1 phase as being distorted cubic with a primitive lattice. Because of certain line splitting in the X-ray pattern, they infer that the material may actually have rhombohedral symmetry. The two patterns given in table 6 are very similar and obviously represent the same phase; neither pattern could be indexed in the present work. It is evident, from the present X-ray data, that 2Y_2_O_3_·Al_2_O_3_ has low symmetry and cannot be designated as cubic or rhombohedral.

The only A^+3^B^+3^O_3_ type compounds found in the present investigation which do not have the perovskite structure are those which occur in the Eu_2_O_3_-In_2_O_3_, Gd_2_O_3_-In_2_O_3_, and Dy_2_O_3_-In_2_O_3_ systems (designated as I in fig. 1). These 1:1 compounds will be reported on more extensively in a following publication [23]. The Eu_2_O_3_·In_2_O_3_, Gd_2_O_3_·In_2_O_3_, and Dy_2_O_3_·In_2_O_3_ compounds appear to be isostructural, having pseudohexagonal symmetry. The Dy_2_O_3_·In_2_O_3_ compound apparently decomposes between 1600 °C and 1650 °C to a mixture of B- and C-rare earth oxide structure types.

### 4.6. Subsolidus Phase Equilibria Relationships

**Figure 5:**
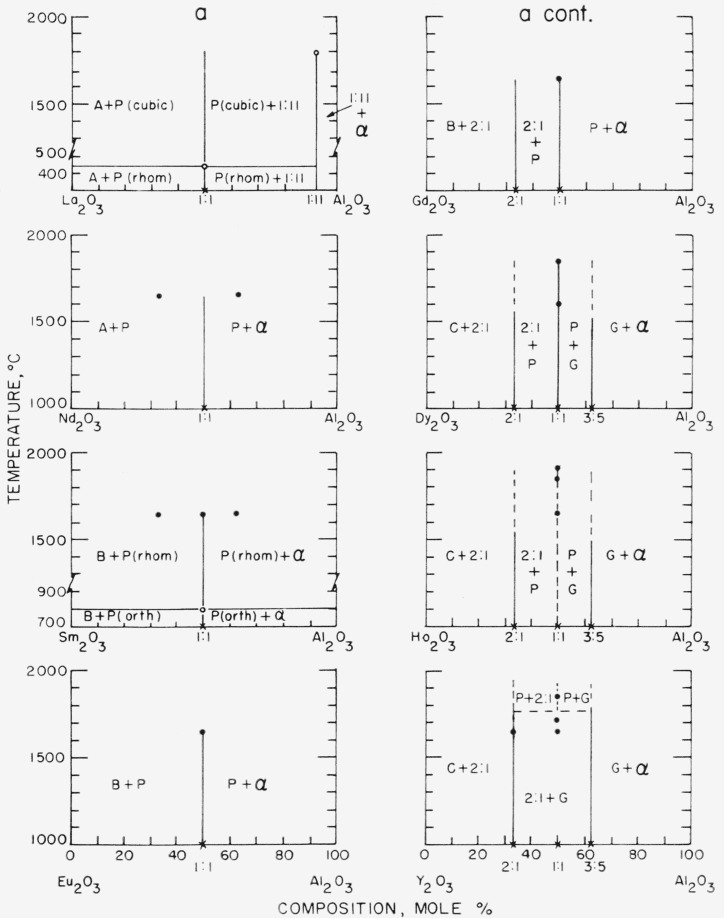

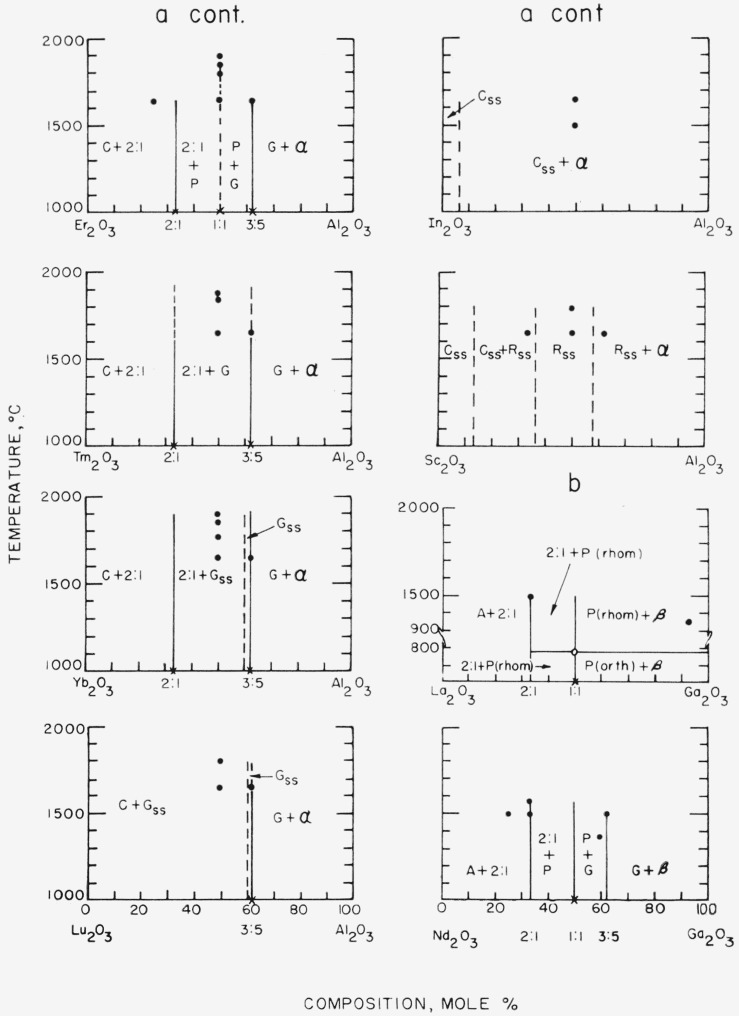

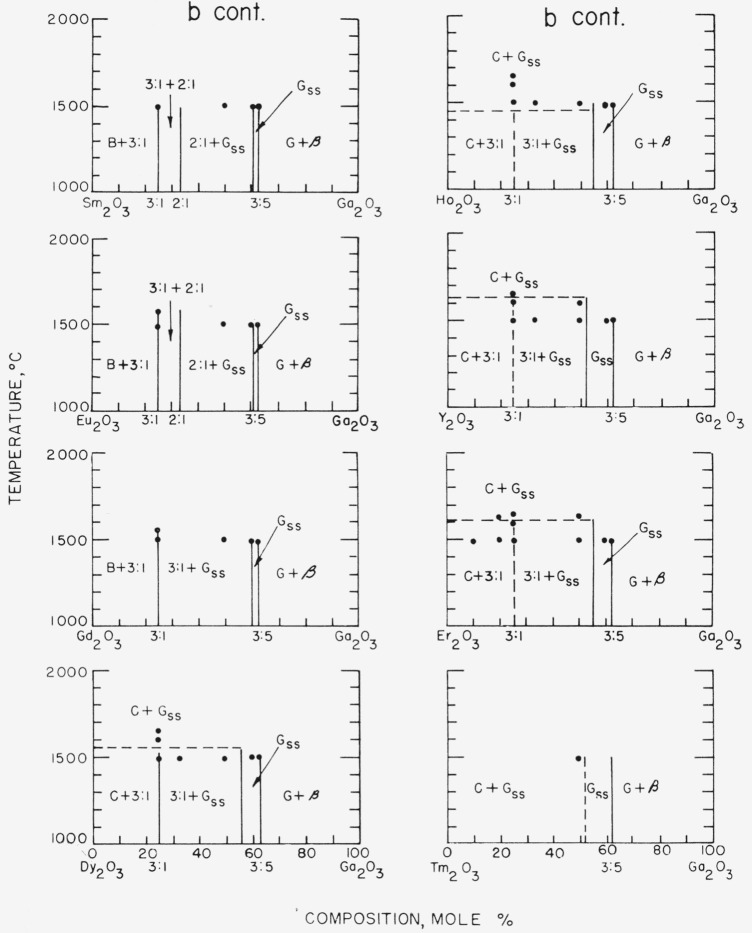

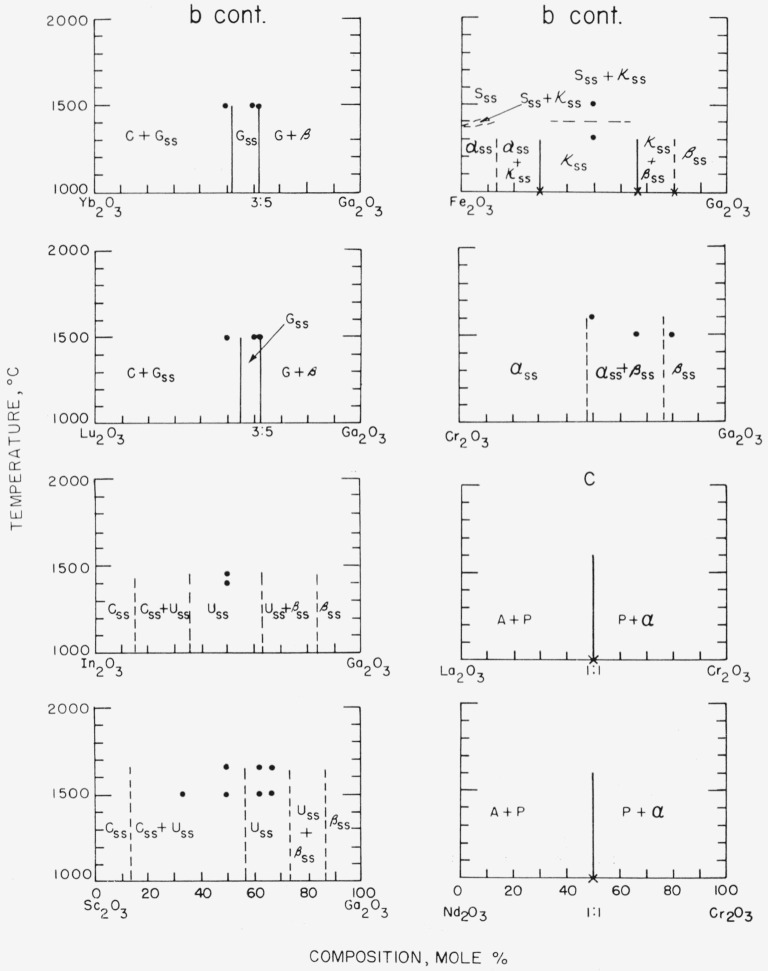

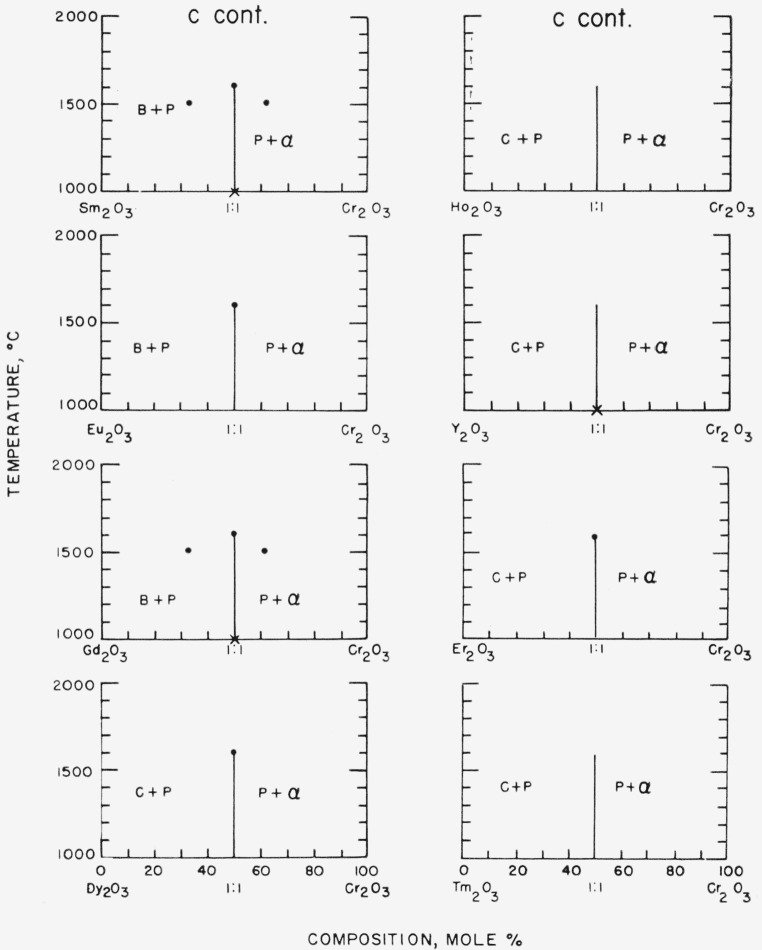

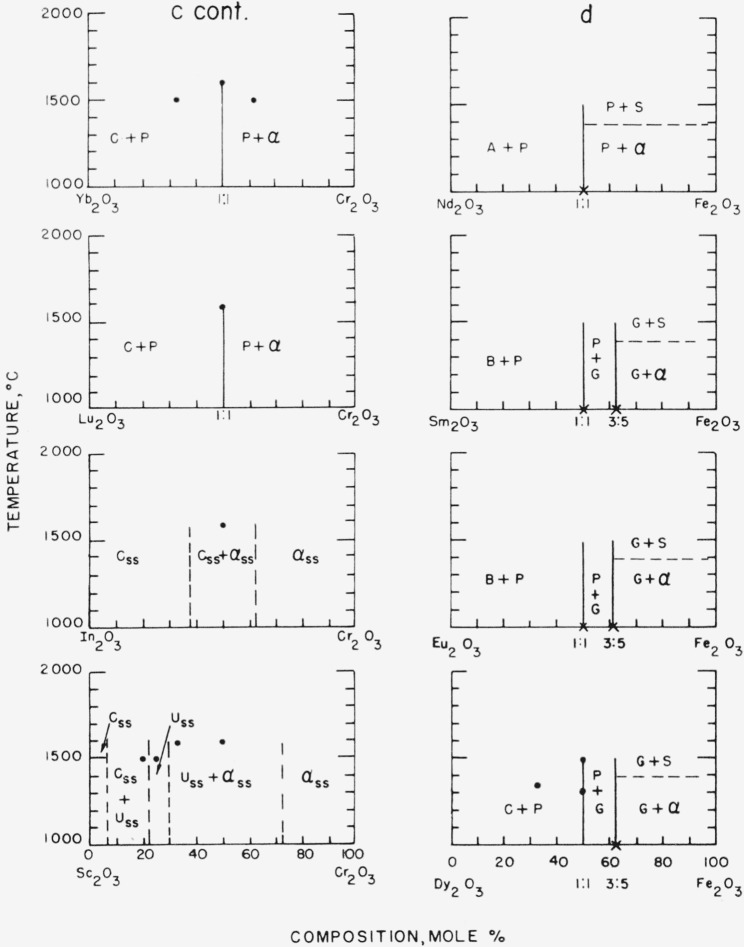

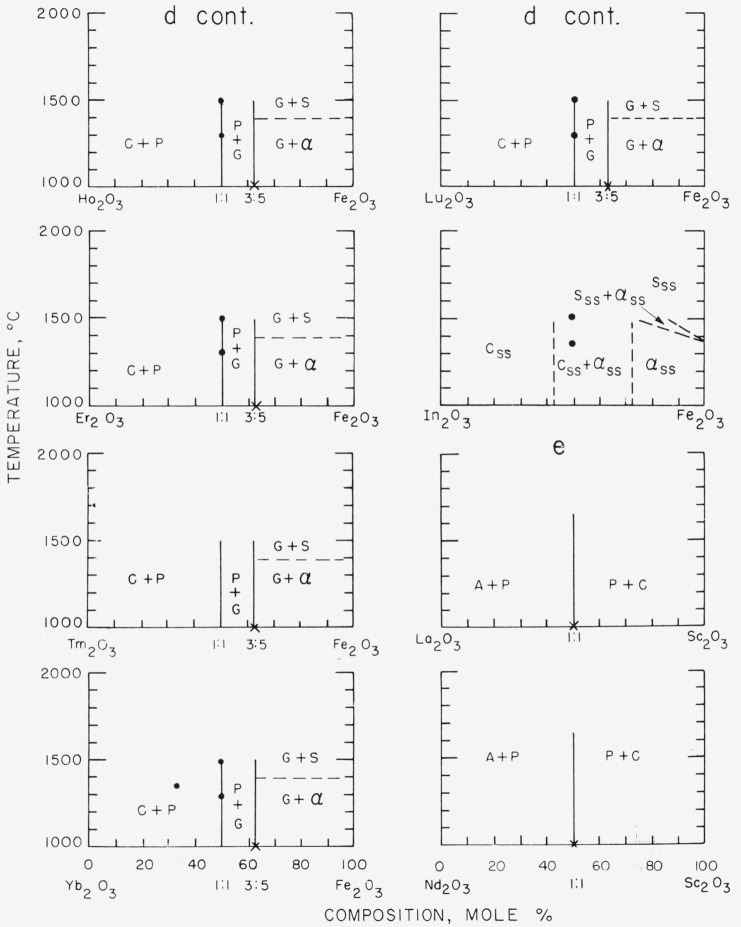

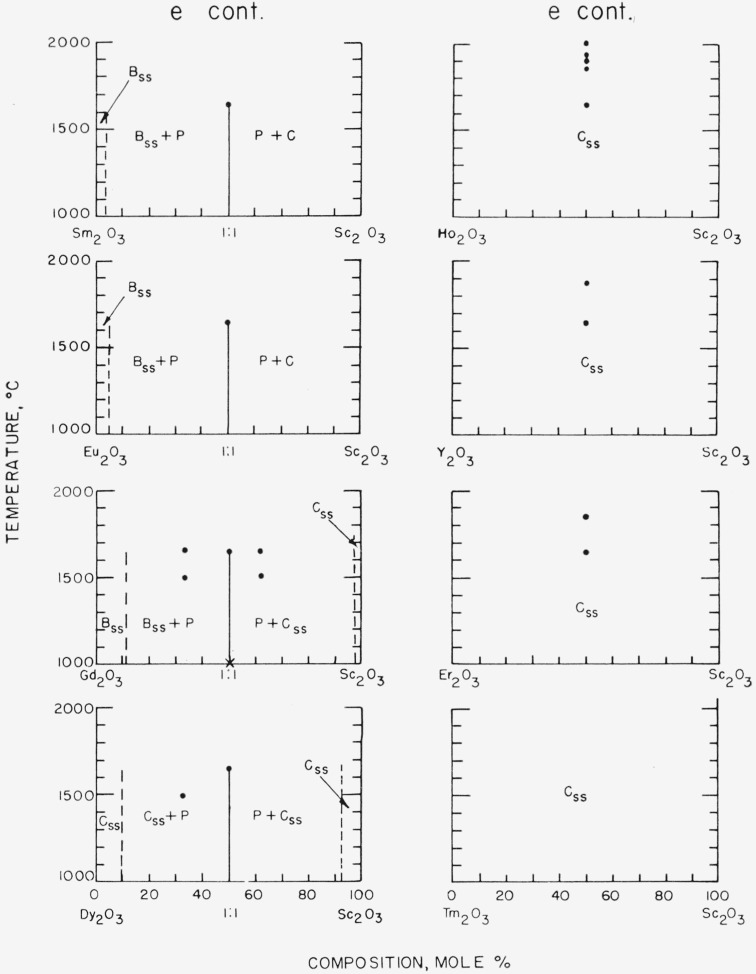

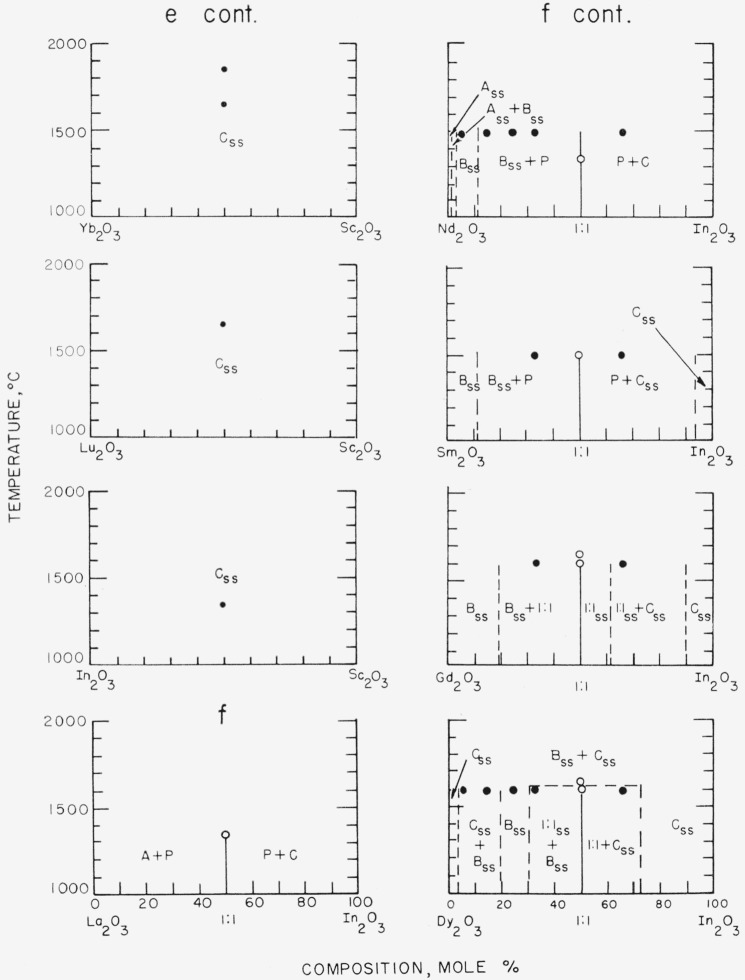

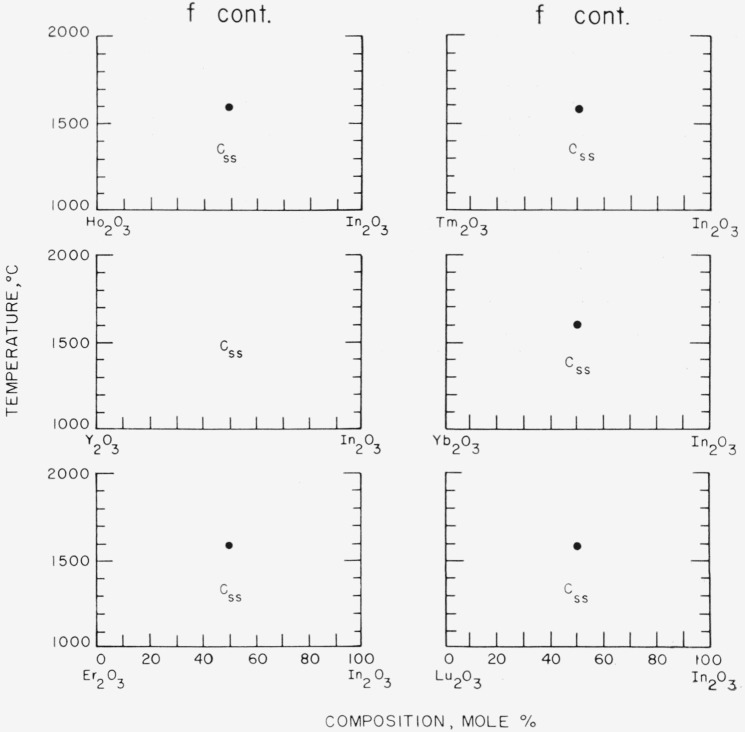
Predicted subsolidus binary phase diagrams for systems involving oxides of the trivalent cations ●—compositions studied in present work ○—data taken from literature X—data taken from literature for which no temperature of heat treatment is given A—A-type rare earth oxide structure B—B-type rare earth oxide structure C—C-type rare earth oxide structure G—garnet type compound 1:11—beta alumina type structure P—perovskite type compound R—unknown type structure, rhombohedral symmetry S—spinel type structure *α*—corundum type structure *β*—beta gallia type structure K—kappa alumina type structure u—unknown type structure similar to kappa alumina ss—solid solution
Binary oxide systems containing Al^+3^ and larger cations. The following systems pertinent to this series have been previously published.
Y_2_O_3_-Al_2_O_3_ [22]Fe_2_O_3_-Al_2_O_3_ [10]Cr_2_O_3_-Al_2_O_3_ [26]Ga_2_O_3_-Al_2_O_3_ [27]Binary oxide systems containing Ga^+3^ and larger cations. The In_2_O_3_-Ga_2_O_3_ system has been previously published [28]. Boundary limits of kappa alumina solid solution taken from Remeika [35].Binary oxide systems containing Cr^+3^ and larger cations. The Fe_2_O_3_-Cr_2_O_3_ system has been previously published [10].Binary oxide systems containing Fe^+3^ and larger cations. The transformation temperature of corundum to spinel type is taken as 1390 °C. [10]. The following systems pertinent to this series have been previously published:
La_2_O_3_-Fe_2_O_3_ [29]Gd_2_O_3_-Fe_2_O_3_ [22]Y_2_O_3_-Fe_2_O_3_ [18]Sc_2_O_3_-Fe_2_O_3_ [30Binary oxide systems containing Sc^+3^ and larger cations.Binary oxide systems containing In^+3^ and larger cations. The Eu_2_O_3_-In_2_O_3_ system to be published [23].

Figure 5 gives the subsolidus phase equilibria relationships for various binary combinations of oxides of the trivalent cations. The figure is divided into six groups of diagrams, each having either Al_2_O_3_, Ga_2_O_3_, Cr_2_O_3_, Fe_2_O_3_, Sc_2_O_3_, or In_2_O_3_ as one component. Previously published diagrams pertinent to a given series are not reproduced here but are included as literature references in the legend of the figure.

All the diagrams were drawn primarily from the data contained in table 1. Data points are indicated by circles on the diagrams. In some instances entire diagrams, or portions thereof, were estimated from the phase relations of similar known systems. The boundaries of the garnet solid solutions were determined by the parametric method while the solid solution areas of A-, B-, or C-type phases were established by a variation of this method as previously described [1]. The boundaries of most of the other type solid solution areas were approximated from X-ray patterns on the basis of the relative amounts of each phase present in a specimen containing two phases. Possible variations of solid solubility with temperature have been ignored in this work. In general, the diagrams must be considered as approximate and minor shifts in solid solution boundary limits may be expected.

The subsolidus phase diagram for the Y_2_O_3_-Al_2_O_3_ system has been included in figure 5a although the diagram has been previously published by Warshaw and Roy [22]. The present diagram differs from the previous one in that a 1:1 perovskite type compound is shown to have a region of stability at elevated temperatures. At lower temperatures, the compound apparently decomposes to a mixture of 2Y_2_O_3_·Al_2_O_3_ and 3Y_2_O_3_·5Al_2_O_3_. The present work does not contradict the published data, but merely extends to temperature ranges not previously reported. A complete reinvestigation of this system is now being undertaken. Because the stability of the 1:1 compound in the Y_2_O_3_-Al_2_O_3_ system is still unknown, the stability of the perovskite phase in the related systems of Ho_2_O_3_-Al_2_O_3_ and Er_2_O_3_-Al_2_O_3_ is also in doubt.

Perhaps one of the more interesting systems investigated is that of Dy_2_O_3_-In_2_O_3_, figure 5f. The phase diagram of this system indicates a solid solution area of B-type rare earth oxide. Since Dy_2_O_3_ and In_2_O_3_ both have the C-type structure, it is unusual for a B-type structure to occur. The largest average cation radius of the B-type solid solution in the Dy_2_O_3_-In_2_O_3_ system is about 0.87 A. This value is appreciably smaller than the radius of Gd^+3^ (0.97 A) which is the smallest rare earth ion to form a pure B-type oxide. Goldschmidt et al. [24] reported that Dy_2_O_3_ formed a B-type structure at elevated temperatures but his work has not yet been confirmed [9]. The formation of the solid solution area of B-type in the Dy_2_O_3_-In_2_O_3_ system might actually indicate that Dy_2_O_3_ does transform from C-to B-type in the pure state. Specimens of Dy_2_O_3_ heated above the melting point of platinum shattered in a manner indicative of a possible reversible phase transformation.

## 5. Summary

A survey was made of the subsolidus reactions that occur in various binary systems involving oxides of the trivalent cations. Incorporated into the study were Al_2_O_3_, Ga_2_O_3_, Cr_2_O_3_, Fe_2_O_3_, Sc_2_O_3_, In_2_O_3_, Y_2_O_3_, and most of the trivalent rare earth oxides. Mixtures in 69 different binary systems were investigated. Specimens were heated at various temperatures until equilibrium was attained and then examined at room temperature by X-ray powder diffraction techniques.

According to the radii of constituent cations, a classification was made of the structure types of the various phases found for equimolar mixtures. The classification consists of a plot of the radii of A^+3^ cations versus the radii of B^+3^ cations and shows specific regions of stability for the different structure types. The graph is divided into regions of one and two phase areas and represents, in addition to several unknown types, the following structures: A-, B-, and C-type rare earth oxide; corundum; beta gallia; kappa alumina; garnet; and perovskite. The classification essentially summarizes the structure types found in all possible binary mixtures of oxides of the trivalent cations studied.

With one exception, all the A^+3^B^+3^O_3_ type compounds which occur have the perovskite structure. The minimum tolerance factors of the alumina and gallia series of perovskite compounds are significantly larger than the Cr_2_O_3_, Fe_2_O_3_, Sc_2_O_3_, and In_2_O_3_ series. The appreciable difference in minimum tolerance factor apparently can be related to the effect of partial covalent bonding.

Appreciable solid solution of the garnet type compounds occurs in binary systems containing Ga_2_O_3_. The range of solid solution generally increases to a maximum at about Tm^+3^ as the radii of the rare earth cation decreases. Solid solution of the garnet compound does not occur in binary systems containing a stable perovskite phase.

Based on the similarity of X-ray patterns, the structure of kappa alumina appears to be related to the structures of (1−*x*)Fe_2_O_3_·*x*Al_2_O_3 ss_ and (1−*x*) Fe_2_O_3_·*x*Ga_2_O_3 ss_. There is also a similarity between these phases and other solid solution phases which occur in the In_2_O_3_-Ga_2_O_3_, Sc_2_O_3_-Ga_2_O_3_, and Sc_2_O_3_-Cr_2_O_3_ systems.

A 2:1 compound occurs in a number of those systems containing either Al_2_O_3_ or Ga_2_O_3_ as one component. A 3:1 compound occurs exclusively in systems containing Ga_2_O_3_. The structures of the 2:1 and 3:1 compounds were not related to any known structure type. A rhombohedral phase which occurs stably in the Sc_2_O_3_-Al_2_O_3_ system and metastably in the In_2_O_3_-Al_2_O_3_ system may have either a fluorite, Sb_2_O_3_, pyrochlore, or C-type structure.

The subsolidus phase assemblages for 79 binary systems were predicted from the data compiled in this investigation.

## Figures and Tables

**Table 1:** Binary oxide mixtures of the trivalent cations AL^+3^ and larger cations

System	Composition	Heat^1^ treatment		Phases identified by X-ray diffraction	Structure type	Symmetry	Unit cell dimensions				Remarks	References^2^
		Temp.	Time				a	b	c	*α*		
												
	*Mole %*	*°C*	*hr*				*A*	*A*	*A*	*deg*		
La_2_O_3_–Al_2_O_3_	50:50	……	………	La_2_O_3_·Al_2_O_3_	Perovskite	Rhombohedral	5.357	………	………	60.1	Rhombohedral to cubic transformation occurs at 435±25 °C [7].	Geller and Bala [7].
	8.3:91.7	1800	1	La_2_O_3_·11Al_2_O_3_	Beta alumina	Hexagonal	5.556	………	22.030	………	……	Roth and Hasko [31].
Ce_2_O_3_–Al_2_O_3_	50:50	1600	1	Ce_2_O_3_·Al_2_O_3_	Perovskite	Rhombohedral	3.766	……	……	90.2	Unit cell dimensions based on face-centered pseudo cell.	Roth [4].
Pr_2_O_3_–Al_2_O_3_	50:50	………	……	Pr_2_O_3_·Al_2_O_3_	Perovskite	Rhombohedral	5.307	………	………	60.33	……	Geller and Bala [7].
Nd_2_O_3_–Al_2_O_3_	66.7:33.3	1350	6	……………	………	……	…………	…………	…………	…………	………	
		1500	6	……	……	……………	………	………	…………	………	……	
		1650	6	Nd_2_O_3_+Nd_2_O_3_·Al_2_O_3_	A-type + perovskite	Hexagonal+rhombohedral.	…………	………	………	…………	……………	
	50:50	………	……	Nd_2_O_3_·Al_2_O_3_	Perovskite	Rhombohedral	5.286	…………	…………	60.42	…………	Geller and Bala [7].
	37.5:62.5	1350	6	……………	……	…………	……	………	…………	……	…………	
		1500	6	………	………	……………	……	…………	………	………	……	
		1650	6	Nd_2_O_3_·Al_2_O_3_	Perovskite	Rhombohedral	………	………	……	………	Al_2_O_3_ not detected; equilibrium phases probably Nd_2_O_3_·Al_2_O_3_+Al_2_O_3_.	
Sm_2_O_3_–Al_2_O_3_	66.7:33.3	1350	6	……………	…………	………	………	………	……	………	………	
		1500	6	…………	………	……………	………	………	………	……	……………	
		1650	6	Sm_2_O_3_+Sm_2_O_3_·Al_2_O_3_	B-type+perovskite	Monoclinic+orthorhombic.	……	………	………	……	…………	
	50:50	……	………	Sm_2_O_3_·Al_2_O_3_	Perovskite	Orthorhombic	5.285	5.290	7.473	………	Results confirmed in present work for specimen heat treated at 1650 °C.	Geller and Bala [7].
											Orthorhombic to rhombohedral transformation occurs at 800 °C [6].	Geller [6].
	37.5:62.5	1350	6	…………	……………	…………	………	……	………	……	…………	
		1650	6	Sm_2_O_3_·Al_2_O_3_	Perovskite	Orthorhombic	……	……	………	………	Al_2_O_3_ not detected; equilibrium phases probably Sm_2_O_3_·Al_2_O_3_+Al_2_O_3_.	
Eu_2_O_3_–Al_2_O_3_	50:50	………	……	Eu_2_O_3_·Al_2_O_3_	Perovskite	Orthorhombic	5.271	5.292	7.458	……	Results confirmed in present work for specimen heat treated at 1650 °C.	Geller and Bala [7].
Gd_2_O_3_–Al_2_O_3_	66.7:33.3	……	……	2Gd_2_O_3_·Al_2_O_3_	Unknown	Unknown	………	……	………	……	The 2:1 phase also forms in the following systems: Dy_2_O_3_–Al_2_O_3_, Ho_2_O_3_–Al_2_O_3_, Y_2_O_3_–Al_2_O_3_, Er_2_O_3_–Al_2_O_3_, Tm_2_O_3_–Al_2_O_3_ and Yb_2_O_3_–Al_2_O_3_ [32].	Warshaw and Roy [32].
	50:50	……	……	Gd_2_O_3_·Al_2_O_3_	Perovskite	Orthorhombic	5.247	5.304	7.417		Results confirmed in present work for specimen heat treated at 1650 °C.	Geller and Bala [7].
	37.5:62.5	1350	6	…………	………	……	………	………	……	………	……	
		1650	6	Gd_2_O_3_·Al_2_O_3_+Al_2_O_3_	Perovskite+corundum.	Orthorhombic+rhombohedral.	……	………	……	……	……………	
Dy_2_O_3_–Al_2_O_3_	50:50	1350	6	…………	…………	……………	………	………	………	……	…………	
		1650	6	Dy_2_O_3_·Al_2_O_3_+3Dy_2_O_3_·5Al_2_O_3_	Perovskite+garnet	Orthorhombic+cubic.	……	……	……	……	Nonequilibrium	
		1850	.17	Dy_2_O_3_·Al_2_O_3_	Perovskite	Orthorhombic	5.21	7.38	5.31	………	……	
	37.5:62.5	………	……	3Dy_2_O_3_·5Al_2_O_3_	Garnet	Cubic	………	……	……	………	Garnet phase (3:5) also forms in the following systems: Tb_2_O_3_–Al_2_O_3_, Ho_2_O_3_–Al_2_O_3_, Y_2_O_3_–Al_2_O_3_, Er_2_O_3_–Al_2_O_3_, Tm_2_O_3_–Al_2_O_3_, Yb_2_O_3_–Al_2_O_3_ and Lu_2_O_3_–Al_2_O_3_ [32].	Warshaw and Roy [32].
H0_2_O_3_–Al_2_O_3_	50:50	1350	6	…………	……………	……………	………	……	………	……	………	
		1650	6	2Ho_2_O_3_·Al_2_O_3_+Ho_2_O_3_·Al_2_O_3_+3Ho_2_O_3_·5A1_2_O_3_	Unknown+perovskite+garnet.	Unknown+orthorhombic++cubic.	……	………	……	………	Nonequilibrium	
		1850	.17	do	do	do	……	………	………	………	Nonequilibrium; 2Ho_2_O_3_·Al_2_O_3_ and 3Ho_2_O_3_·5Al_2_O_3_ decreased in amounts relative to previous heat.	
		1910	.5	Ho_2_O_3_·A1_2_O_3_	Perovskite	Orthorhombic	5.18	7.36	5.33	……	……	
Y_2_O_3_–Al_2_O_3_^3a^	66.7:33.3	1100	16	…………	…………	………	………	……	………	………	……	
		1650	6	2Y_2_O_3_·Al_2_O_3_	Unknown	Unknown	………	……	………	………	………	
	50:50	……	……	Y_2_O_3_·Al_2_O_3_	Perovskite	Orthorhombic	5.179	5.329	7.370	………	………	Geller and Bala [7].
		1350	6	…………	……………	……………	……	………	……	………	………	
		1500	6	Y_2_O_3_+2Y_2_O_3_·Al_2_O_3_+3Y_2_O_3_·5Al_2_O_3_+Y_2_O_3_·Al_2_O_3_	C–type+unknown+garnet+perovskite.	Cubic+unknown+cubic+orthorhombic.	……	………	………	……	Nonequilibrium	
		1850	.5	Y_2_O_3_·Al_2_O_3_	Perovskite	Orthorhombic	………	………	……	……	…………	
		1705	.5	Y_2_O_3_·Al_2_O_3_+2Y_2_O_3_·Al_2_O_3_+3Y_2_O_3_·5Al_2_O_3_	Perovskite+unknown+garnet.	Orthorhombic+unknown+cubic.	………	……	……	………	Perovskite apparently decomposing; nonequilibrium.	
		1645	1	do	do	do	………	………	……	……	Garnet and 2:1 phases increased in amount relative to previous heat; nonequilibrium.	
	37.5:62.5	1650	2	3Y_2_O_3_·5Al_2_O_3_	Garnet	Cubic	12.01	………	……	……	…………	Yoder and Keith [33].
		1730	1	do	do	do	……	……	……	……	……………	
Er_2_O_3_–Al_2_O_3_	75:25	1350	6	………	………	………	………	……	……	………	………	
		1500	6	………	………	………	……	……	………	……	……	
		1650	6	Er_2_O_3_+2Er_2_O_3_·Al_2_O_3_	C–type+unknown	Cubic+unknown	……	……	……	……	………	
	50:50	1350	6	…………	………	…………	……	………	……	……	……	
		1650	6	Er_2_O_3_+2Er_2_O_3_·Al_2_O_3_+Er_2_O_3_·Al_2_O_3_+3Er_2_O_3_·5Al_2_O_3_.	C–type+unknown+perovskite+garnet.	Cubic+unknown+orthorhombic+cubic.	11.983	………	………	………	Nonequilibrium; listed dimension for garnet phase in mixture.	
		1800	.5	do	do	…………	……	……	……	……	Nonequilibrium; no apparent change in relative amounts from previous heat.	
		1350	6	…………	……………	………	……	………	……	………	……	
		1650	6	………	…………	…………	………	……	……	……	………	
		1850	6	do	do	do	……	……	……	………	Nonequilibrium; C–type, 2:1 and garnet phases reduced in amount relative to 1650 °C heat.	
		1350	6	………	…………	…………	………	……	………	………	……………	
		1650	6	………	………	…………	……	……	………	………	…………	
		1900	.75	Er_2_O_3_·Al_2_O_3_	Perovskite	Orthorhombic	………	………	………	………	Specimen melted	
		1350	6	………	…………	…………	………	……	………	………	………	
		1650	6	……	………	………	………	……	………	……	………	
		1910	.08	…………	………	………	……	……	……	……	……	
		1840	.75	Er_2_O_3_·Al_2_O_3_	Perovskite	Orthorhombic	5.16	7.33	5.32	……	Specimen melted; then annealed	
	37.5:62.5	1350	6	…………	………	………	………	……	……	………	…………	
		1600	6	………	………	………	……	………	………	……	……	
		1650	12	Er_2_O_3_·Al_2_O_3_+3Er_2_O_3_·5Al_2_O_3_	Perovskite+garnet	Orthorhombic+cubic	11.983	……	……	……	Nonequilibrium; listed dimension for garnet phase in mixture.	
Tm_2_O_3_-–Al_2_O_3_	50:50	1350	6	…………	…………	…………	………	……	……	………	…………	
		1650	6	2Tm_2_O_3_·Al_2_O_3_+Tm_2_O_3_·Al_2_O_3_+3Tm_2_O_3_·5Al_2_O_3_.	Unknown+perovskite+garnet.	Unknown+orthorhombic+cubic.	5.15 11.96	7.29	5.33	………	Nonequilibrium; listed dimensions for perovskite and garnet phases in mixture.	
		1850	.7	do	do	do	………	……	………	………	Nonequilibrium; no apparent change in relative amounts from previous heats.	
		1350	6	…………	…………	…………	………	………	……	………	………	
		1650	6	…………	………	…………	………	………	……	……	………	
		1870	.08	………	………	……	………	………	………	………	………	
		1845	.25	2Tm_2_O_3_·Al_2_O_3_+Tm_2_O_3_·Al_2_O_3_+3Tm_2_O_3_·5Al_2_O_3_.	Unknown+perovskite+garnet.	Unknown+orthorhombic+cubic.	……	………	………	………	Specimen melted; then annealed. Nonequilibrium; perovskite considerably reduced in amount compared to 1850 °C specimen.	
	37.5:62.5	1350	6	………	…………	………	………	………	……	………	……	
		1600	6	………	……	……	……	……	………	………	……	
		1650	12	3Tm_2_O_3_·5Al_2_O_3_	garnet	Cubic	11.96	……	……	……	……	
Yb_2_O_3_–Al_2_O_3_	50:50	1350	6		………	……	………	……	………	……	……	
		1650	6	Yb_2_O_3_+2Yb_2_O_3_·Al_2_O_3_+3Yb_2_O_3_·5Al_2_O_3 ss_.	C-type+unknown+garnet.	Cubic+unknown+cubic.	11.946	………	……	………	Nonequilibrium; listed dimension for garnet phase in mixture.	
		1770	0.33	do	do	do	………	………	………	………	Nonequilibrium C-type phase reduced in amount relative to previous heat.	
		1850	.33	do	do	do	………	………	………	……	Nonequilibrium; C-type phase reduced in amount relative to previous heat.	
	37.5:62.5	1350	6	………	………	………	………	………	……	………	………	
		1600	6	………	………	………	……	………	………	………	………	
		1650	12	3Yb_2_O_3_·5Al_2_O_3_.	Garnet	Cubic	11.931	……	………	……	………	
Lu_2_O_3_–Al_2_O_3_	50:50	1350	6	………	……	………	………	……	……	……	………	
		1650	6	Lu_2_O_3_+3Lu_2_O_3_·5Al_2_O_3 ss_.	C-type+garnet	Cubic+cubic	11.927	……	……	………	Listed dimension for garnet phase in mixture.	
		1800	0.17	do	do	do	………	………	………	………	………	
	37.5:62.7	1350	6	………	……………	……	……	……	……	………	……………	
		1600	6	………	………	………	………	………	………	………	………	
		1650	6	3Lu_2_O_3_·5Al_2_O_3_	Garnet	Cubic	11.912	……	……	……	May be some 2:1 phase present.	
In_2_O_3_·Al_2_O_3_	50:50	800	20	……	…………	………	………	………	………	………	…………	
		1350	6	………	………	………	………	………	……	………	…………	
		1500	46	In_2_O_3 ss_+Al_2_O_3_	C-type+corundum	Cubic+rhombohedral	……	………	……	………	………	
		1650	42	do	do	do	……	………	……	………	………	
		800	20	……	………	………	……	……	……	………	………	
		1350	6	………	………	………	………	………	………	……	………	
		1700	0.0	In_2_O_3 ss_+unknown	C-type+unknown	Cubic+unknown	……	……	………	……	Specimen melted, unidentified phase appears to be isostructural with rhombohedral phase of Sc_2_O_3_–Al_2_O_3_ system. This phase similar to unknown phase previously reported by Keith and Roy [3].	
Sc_2_O_3_–Al_2_O_3_	66.7:33.3	1350	6	………	………	………	……	……	………	………	………	
		1650	6	Sc_2_O_3 ss_+(1−*x*) Sc_2_O_3_·*x*Al_2_O_3 ss_.	C-type+unknown	Cubic+rhombohedral	………	………	……	………	……	
	50:50	1350	6	………	…………	…………	………	………	………	………	………	
		1650	9.5	(1−*x*)Sc_2_O_3_·*x*Al_2_O_3 ss_.	Unknown	Rhombohedral	9.45	………	……	87.4	Structure type appears to be rhombohedral distortion of fluorite type; see text.	
		1790	0.08	do	do	do	……	………	……	………	………	
		1960	.08	do	do	do	………	………	……	………	Specimen melted	
Fe_2_O_3_–Al_2_O_3_^3b^	53:47	800	20	………	………	……	……	………	………	……	………	
		1000	65	………	………	………	………	………	………	………	……	
		1375	49	(1−*x*) Fe_2_O_3_·*x*Al_2_O_3 ss_	Kappa alumina	Orthorhombic	………	………	……	……	……	
	50:50	800	20		………	…………	………	………	………	………	………	
		1000	65	………	………	………	………	………	……	………	……	
		1350	46	(1−*x*) Fe_2_O_3_·*x*Al_2_O_3 ss_	Kappa alumina	Orthorhombic	………	……	……	………	…………	
		1450	………	(1−*x*) Fe_2_O_3_·*x*Al_2_O_3 ss_	Kappa alumina	Orthorhombic	7.03	6.33	7.411	………	This cell apparently not correct; see text.	Richardson, Ball and Rigby [20].
	47:53	800	20	………	………	………	……	……	………	………	…………	
		1000	65	…………	…………	………	……	………	………	……	………	
		1350	46	(1−*x*) Fe_2_O_3_·*x*Al_2_O_3 ss_	Kappa alumina	Orthorhombic	8.59	9.23	4.98	……	………	
Cr_2_O_3_–Al_2_O_3_^3c^	50:50	1000	6	………	………	………	………	………	……	……	………	
		1600	6	(1−*x*) Cr_2_O_3_·*x*Al_2_O_3 ss_	Corundum	Rhombohedral	……	……	………	………	………	
Ga_2_O_3_–Al_2_O_3_^4d^	50:50	1350	6	…………	…………	………	……	………	………	………	……	
		1650	6	Ga_2_O_3 ss_	Beta gallia	Monoclinic	……	………	………	………	………	
	30:70	1350	6	……	………	………	………	………	……	………	………	
		1650	6	Ga_2_O_3 ss_+Al_2_O_3 ss_	Beta gallia+Corundum.	Monoclinic+rhombohedral.	……	……	………	………	………	
**Ga^+3^ and larger cations**												
La_2_O_3_–Ga_2_O_3_	66.7:33.3	1350	6	………	………	………	………	………	………	………	……	
		1500	42	2La_2_O_3_·Ga_2_O_3_	Unknown	Unknown	………	………	……	………	May be small amount of perovskite type compound present.	
	50:50	………	……	La_2_O_3_·Ga_2_O_3_	Perovskite	Orthorhombic	5.496	5.524	7.787	……	Orthorhombic to rhombohedral transformation occurs at 875 °C [6].	Geller [6].
	8.2:91.8	1000	20	………	……	……	………	………	………	………	……	
		1350	6	La_2_O_3_·Ga_2_O_3_+Ga_2_O_3_	Perovskite+beta gallia	Orthorhombic+monoclinic.	……	………	………	………	………	
Pr_2_O_3_–Ga_2_O_3_	50:50	……	……	Pr_2_O_3_·Ga_2_O_3_	Perovskite	Orthorhombic	5.465	5.495	7.729	……	…………	Geller [6].
Nd_2_O_3_–Ga_2_O_3_	75:25	1350	6	………		…………	……	………	……	………	……	
		1500	6	Nd_2_O_3_+2Nd_2_O_3_·Ga_2_O_3_	A-type+unknown	Hexagonal+unknown.	………	………	……	……	Specimen partially melted	
	66.7:33.3	1350	6	………	………	………	……	……	……	………	………	
		1500	6	Nd_2_O_3_+2Nd_2_O_3_·Ga_2_O_3_+Nd_2_O_3_·Ga_2_O_3_.	A-Type+unknown+perovskite.	Hexagonal+unknown+orthorhombic.	………	………	………	……	Nonequilibrium	
		1575	6	2Nd_2_O_3_·Ga_2_O_3_	Unknown	Unknown	………	……	……	……	………	
	50:50	………	………	Nd_2_O_3_·Ga_2_O_3_	Perovskite	Orthorhombic	5.426	5.502	7.706	……	………	Geller [6].
	40:60	1000	6	……	………	………	………	……	……	……	………	
		1350	46	Nd_2_O_3_·Ga_2_O_3_+3Nd_2_O_3_·5Ga_2_O_3_	Perovskite+garnet	Orthorhombic+cubic	12.505	………	………	………	Listed dimension for garnet phase in mixture.	
	37.5:62.5	1000	6	……	………	………	………	……	……	……	……	
		1350	6	………	……	……	………	………	………	………	……	
		1500	46	3Nd_2_O_3_·5Ga_2_O_3_	Garnet	Cubic	12.505	……	………	………	……	
Sm_2_O_3_–Ga_2_O_3_	75:25	1350	6	……	……	……	………	……	……	……	………	
		1500	6	3Sm_2_O_3_·Ga_2_O_3_+2Sm_2_O_3_·Ga_2_O_3_	Unknown+unknown	Unknown+unknown	………	………	……	……	Nonequilibrium	
		1575	6	3Sm_2_O_3_·Ga_2_O_3_	Unknown	Unknown	………	………	……	………	……	
	50:50	1350	6	……	……	………	………	………	……	………	………	
		1500	6	2Sm_2_O_3_·Ga_2_O_3_+3Sm_2_O_3_·5Ga_2_O_3 ss_	Unknown+garnet	Unknow+cubic	12.448	………	………	………	Listed dimension for garnet phase in mixture.	
	40:60	1000	20	………	………	………	………	……	………	……	………	
		1350	6	………	……	………	……	………	………	………	……	
		1500	44	2Sm_2_O_3_·Ga_2_O_3_+3Sm_2_O_3_·5Ga_2_O_3 ss_	Unknown+garnet	Unknown+cubic	12.448	……	………	……	Listed dimension for garnet phase in mixture.	
	37.5:62.5	1000	6	………	………	………	………	………	………	………	………	
		1350	6		………	……	………	………	……	……	………	
		1500	46	3Sm_2_O_3_·5Ga_2_O_3_	Garnet	Cubic	12.434	………	……	………	……	
Eu_2_O_3_–Ga_2_O_3_	75:25	1350	6	………	……	………	………	………	………	………	………	
		1500	6	3Eu_2_O_3_·Ga_2_O_3_+2Eu_2_O_3_·Ga_2_O_3_	Unknown+unknown	Unknown+unknown	………	………	………	………	………	
		1575	6	3Eu_2_O_3_·Ga_2_O_3_	Unknown	Unknown	………	………	……	……	………	
	50:50	1350	6	………	………	………	……	……	……	……	……	
		1500	6	2Eu_2_O_3_·Ga_2_O_3_+3Eu_2_O_3_·5Ga_2_O_3 ss_	Unknown +garnet	Unknown+Cubic	12.431	……	………	………	Listed dimension for garnet phase in mixture.	
	40:60	1000	6	………	……	………	……	………	………	………	……	
		1350	6	………	………	………	………	……	………	………	……	
		1500	44	3Eu_2_O_3_·5Ga_2_O_3 ss_	Garnet	Cubic	12.422	………	……	……	……	
	37.5:62.5	1000	6		……	………	……	………	……	……	……	
		1350	6	………	……	………	………	……	……	………	……	
		1500	46	3Eu_2_O_3_·5Ga_2_O_3_	Garnet	Cubic	12.403	………	………	……	……	
Gd_2_O_3_–Ga_2_O_3_	75:25	1350	6	………	………	……	………	………	………	………	………	
		1500	6	3Gd_2_O_3_·Ga_2_O_3_	Unknown	Unknown	……	………	………	………	……	
		1600	6	3Gd_2_O_3_·Ga_2_O_3_	Unknown	Unknown	………	………	……	………	……	
	50:50	1350	6	………	………	……	………	………	………	……	………	
		1500	6	3Gd_2_O_3_·Ga_2_O_3_+3Gd_2_O_3_·5Ga_2_O_3 ss_	Unknown+garnet	Unknown+cubic	12.426	……	………	………	Listed dimension for garnet phase in mixture.	
	40:60	1000	6	………	………	………	……	……	………	………	………	
		1350	6	………	……	……	………	………	……	………	………	
		1500	44	3Gd_2_O_3_·5Ga_2_O_3 ss_	Garnet	Cubic	12.416	……	………	………	………	
	37.5:62.5	1000	6	……	……	……	………	……	……	………	……	
		1350	6	……	……	……	……	………	………	………	………	
		1500	46	3Gd_2_O_3_·5Ga_2_O_3_	Garnet	Cubic	12.377	……	………	……	……	
Dy_2_O_3_–Ga_2_O_3_	75:25	1350	6	……	………	………	………	………	………	………	………	
		1500	6	3Dy_2_O_3_·Ga_2_O_3_	Unknown	Unknown	………	……	………	………	………	
		1600	6	Dy_2_O_3_+3Dy_2_O_3_·Ga_2_O_3_	C-type+unknown	Cubic+unknown	……	………	……	……	Nonequilibrium; the 3:1 phase apparently is decomposing.	
		1650	6	do	do	do	………	……	……	………	………	
	66.7:33.3	1350	6	……	……	……	………	………	……	………	………	
		1500	6	3Dy_2_O_3_·Ga_2_O_3_+3Dy_2_O_3_·5Ga_2_O_3_	Unknown+garnet	Unknown+cubic	……	……	………	………	………	
	50:50	1350	6	……	………	………	………	………	………	………	………	
		1500	6	3Dy_2_O_3_·Ga_2_O_3_+3Dy_2_O_3_·5Ga_2_O_3 ss_	Unknown+garnet	Unknown+cubic	12.417	………	………	……	Listed dimension for garnet phase in mixture	
	40:60	1000	6	………	………	……	……	………	………	……	……	
		1350	6	……	……	……	……	………	………	………	………	
		1500	44	3Dy_2_O_3_·5Ga_2_O_3 ss_	Garnet	Cubic	12.349	……	……	………	………	
	37.5:62.5	1000	6	……	………	………	……	………	………	………	………	
		1350	6	……	…………	…………	………	……	…………	…………	………	……
		1500	46	3Dy_2_O_3_·5Ga_2_O_3_	Garnet	Cubic	12.308	……	……	………	……	
Ho_2_O_3_–Ga_2_O_3_	75:25	1350	6	……	……	……	……	………	……	……	………	
		1500	6	Ho_2_O_3_+3Ho_2_O_3_·Ga_2_O_3_	C-type+unknown	Cubic+unknown	………	………	………	………	Nonequilibrium	
		1600	6	do	do	do	……	……	………	………	do	
		1650	6	do	do	do	………	………	……	………	do	
	66.7:33.3	1350	6	………	……	……	………	………	………	………	………	
		1500	6	3Ho_2_O_3_·Ga_2_O_3_+3Ho_2_O_3_·5Ga_2_O_3 ss_	Unknown+garnet	Unknown+cubic	……	………	………	……	………	
	50:50	1350	6	……	……	………	………	……	………	………	………	
		1500	6	3Ho_2_O_3_·Ga_2_O_3_+3Ho_2_O_3_·5Ga_2_O_3 ss_	Unknown+garnet	Unknown+cubic	12.408	………	………	………	Listed dimension for garnet phase in mixture.	
	40:60	1000	6	………	……	………	………	………	………	……	……	
		1350	6	……	………	………	……	……	………	………	……	
		1500	44	3Ho_2_O_3_·5Ga_2_O_3 ss_	Garnet	Cubic	12.324	……	……	……	………	
	37.5:62.5	1000	6	…………	………	………	………	……	……	……	……	
		1350	6	………	……	………	……	……	……	………	……	
		1500	46	3Ho_2_O_3_·5Ga_2_O_3_	Garnet	Cubic	12.282	……	……	………	……	
Y_2_O_3_–Ga_2_O_3_	75:25	1350	6	…………	………	………	……	……	……	………	……	
		1500	6	Y_2_O_3_+3Y_2_O_3_·Ga_3_O_3_+3Y_2_O_3_·5Ga_2_O_3 ss_	C-type+unknown+garnet.	Cubic+unknown+cubic	………	……	……	………	Nonequilibrium	
		1600	6	do	do	do	……	……	………	………	do	
		1650	6	do	do	do	……	……	……	…………	do	
	66.7:33.3	1350	6	……	……	……	………	………	………	………	………	
		1500	6	3Y_2_O_3_·Ga_2_O_3_+3Y_2_O_3_·5Ga_2_O_3 ss_	Unknown+garnet	Unknown+cubic	……	……	……	………	………	
	50:50	1350	6	………	………	……	………	………	………	………	……	
		1500	6	3Y_2_O_3_·Ga_2_O_3_+3Y_2_O_3_·5Ga_2_O_3 ss_	Unknown+garnet	Unknown+cubic	12.441	………	………	………	Listed dimension for garnet phase in mixture.	
		1600	6	do	do	do	……	……	……	………	……	
	40:60	1000	6	……	……	………	………	………	………	………	………	
		1350	6	………	……	………	………	…………	……	……	………	
		1500	44	3Y_2_O_3_·5Ga_2_O_3 ss_	Garnet	Cubic	12.318	………	………	………	………	
	37.5:62.5	1000	6	……	……	………	………	……	………	………	………	
		1350	6	………	………	……	………	………	……	……	………	
		1500	46	3Y_2_O_3_·5Ga_2_O_3_	Garnet	Cubic	12.275	……	………	………	………	
Er_2_O_3_–Ga_2_O_3_	90:10	1350	6	……	……	……	………	………	……	………	………	
		1500	6	Er_2_O_3_+3Er_2_O_3_·Ga_2_O_3_	C-type+unknown	Cubic+unknown	………	………	………	………	………	
	80:20	1350	6	……	………	……	………	………	………	………	………	
		1500	6	Er_2_O_3_+3Er_2_O_3_·Ga_2_O_3_	C-type+unknown	Cubic+unknown	……	……	……	……	………	
			1.3	do	do	do	………	……	………	………	……	
		1350	6	……	………	……	………	………	……	………	………	
		1500	6	……	……	……	………	……	………	……	………	
			.08	………	…………	……	………	………	………	………	………	
		1625	.25	Er_2_O_3_+3Er_2_O_3_·5Ga_2_O_3 ss_	C-type+garnet	Cubic+cubic	………	………	………	………	Specimen melted; then annealed	
	75:25	1350	6	………	………	………	……	……	……	……	………	
		1500	6	Er_2_O_3_+Er_2_O_3_·Ga_2_O_3_+3Er_2_O_3_·5Ga_2_O_3_	C-type+unknown+garnet	Cubic+unknown+cubic	………	………	………	………	Nonequilibrium	
		1600	6	Er_2_O_3_+3Er_2_O_3_·Ga_2_O_3_	C-type+unknown	Cubic+unknown	……	……	……	………	Nonequilibrium; C-type phase considerably reduced in amount relative to previous heat.	
		1650	6	do	do	do	………	……	………	………	Nonequilibrium; C-type phase increased in amount relative to previous heat.	
		1350	6	………	………	………	……	……	……	……	……	
		1500	6	………	………	………	………	………	………	……	……	
		1745	.08	……	……	……	……	……	……	……	……	
		1700	.5	Er_2_O_3_+3Er_2_O_3_·5Ga_2_O_3 ss_	C-type+garnet	Cubic+cubic	………	………	……	………	Specimen melted; then annealed	
	50:50	1350	6	……	……	……	……	……	………	………	………	
		1500	6	3Er_2_O_3_·Ga_2_O_3_+3Er_2_O_3_·5Ga_2_O_3 ss_	Unknown+garnet	Unknown+cubic	12.400	………	………	………	Listed dimension for garnet phase in mixture.	
		1645	.5	do	do	………	……	………	………	……	………	
		1350	6	……	……	……	………	………	………	………	………	
		1500	6	……	……	……	………	………	………	………	………	
		1745	.08	………	……………	………	………	………	………	………	……	
		1635	.25	Er_2_O_3_+3Er_2_O_3_·5Ga_2_O_3 ss_	C-type+garnet	Cubic+cubic	……	………	………	………	Specimen melted; then annealed	
	40:60	1000	6	…………	………	……	………	………	………	………	………	
		1350	6	……	……	………	………	………	………	………	………	
		1500	44	3Er_2_O_3_·5Ga_2_O_3 ss_	Garnet	Cubic	12.300	………	……	………	………	
	37.5:62.5	1000	6	………	………	……	………	………	……	………	………	
		1350	6	……	………	……	………	………	………	………	………	
		1500	46	3Er_2_O_3_·5Ga_2_O_3_	Garnet	Cubic	12.254	………	……	………	………	
Tm_2_O_3_-Ga_2_O_3_	50:50	1350	6	………	………	……	………	………	……	………	………	
		1500	6	Tm_2_O_3_+3Tm_2_O_3_·5Ga_2_O_3 ss_	C-type+garnet	Cubic+cubic	12.369	……	………	………	Listed dimension for garnet phase in mixture.	
Yb_2_O_3_–Ga_2_O_3_	50:50	1350	6	……	………	……	……	……	……	………	……	
		1500	6	Yb_2_O_3_+3Yb_2_O_3_·5Ga_2_O_3 ss_	C-type+garnet	Cubic+cubic	12.344	………	……	………	Listed dimension for garnet phase in mixture.	
	40:60	1000	6	………	………	……	………	………	………	………	………	
		1350	6	……	………	………	………	………	………	………	………	
		1500	44	3Yb_2_O_3_·5Ga_2_O_3 ss_	Garnet	Cubic	12.232	………	………	………	………	
	37.5:62.5	1000	6	………	……	………	………	………	………	………	………	
		1350	6	………	……	………	……	………	………	………	………	
		1500	46	3Yb_2_O_3_·5Ga_2_O_3_	Garnet	Cubic	12.200	………	………	……	………	
Lu_2_O_3_–Ga_2_O_3_	50:50	1350	6	……	………	……	………	………	………	……	………	
		1500	6	Lu_2_O_3_+3Lu_2_O_3_·5Ga_2_O_3 ss_	C-type+garnet	Cubic+cubic	12.320	………	………	………	Listed dimension for garnet phase in mixture.	
	40:60	1000	6	……	……	……	………	………	………	………	………	
		1350	6	……	………	………	……	……	………	……	………	
		1500	44	3Lu_2_O_3_·5Ga_2_O_3 ss_	Garnet	Cubic	12.231	………	………	………	………	
	37.5:62.5	1000	6	……	……	……	………	………	………	……	………	
		1350	6	……	………	……	………	……	………	………	………	
		1500	46	3Lu_2_O_3_·5Ga_2_O_3_	Garnet	Cubic	12.183	………	……	………	………	
In_2_O_3_–Ga_2_O_3_^3e^	50:50	800	20	………	………	……	……	………	………	………	………	
		1400	46	………	……	………	……	………	……	………	………	
		1450	46	(1−*x*)In_2_O_3_·*x*Ga_2_O_3 ss_	Unknown	Unknown	………	………	………	………	Similar to kappa alumina type phases.	
Sc_2_O_3_–Ga_2_O_3_	66.7:33.3	1350	6	………	……	………	………	………	………	………	………	
		1500	6	Sc_2_O_3 ss_+(1−*x*)Sc_2_O_3_·2Ga_2_O_3 ss_	C-type+unknown	Cubic+unknown	………	……	………	………	………	
	50:50	1350	6	………	………	……	………	………	………	………	………	
		1500	6	……	………	………	………	……	………	……	………	
		1650	6	Sc_2_O_3 ss_+(1−*x*)Sc_2_O_3_·*x*Ga_2_O_3 ss_	C-type+unknown	Cubic+unknown	……	………	………	………	……	
	37.5:62.5	1350	6	………	……	……	………	……	………	………	……	
		1500	6	……	……	……	………	……	……	………	………	
		1650	6	(1−*x*)Sc_2_O_3_·*x*Ga_2_O_3 ss_	Unknown	Unknown	………	………	………	………	Unknown phase similar to kappa alumina type phases. It also appears to be isostructural with (1−*x*)In_2_O_3_·*x*Ga_2_O_3 ss_	
	33.3:66.7	1350	6	…………	…………	…………	……	……	……	………	………	
		1500	6	………	………	………	………	………	………	………	………	
		1650	6	(1−*x*)Sc_2_O_3_·*x*Ga_2_O_3 ss_	Unknown	Unknown	………	……	………	………	………	
Fe_2_O_3_–Ga_2_O_3_	~50:50	………	………	(1−*x*)Fe_2_O_3_·*x*Ga_2_O_3 ss_ (*x~.*5)	Unknown	Orthorhombic	8.75	9.40	5.07	………	……	Wood [21].
	50:50	800	20	………	…………	………	………	………	………	………	……	
		1000	65	……	……	……	………	………	……	………	………	
		1300	6	(1−*x*)Fe_2_O_3_·*x*Ga_2_O_3 ss_	Kappa alumina	Orthorhombic	8.73	9.38	5.08	………	Same phase as reported by Wood [2]	
		1500	6	Fe_3_O4 _ss_+Ga_2_O_3 ss_	Spinel+beta gallia	Cubic+monoclinic	……	………	………	………	………	
Cr_2_O_3_–Ga_2_O_3_	50:50	800	6	……	……	……	………	………	………	………	………	
		1000	6	………	……	………	………	………	………	………	………	
		1600	6	Cr_2_O_3 ss_+Ga_2_O_3 ss_	Corundum+beta gallia.	Rhombohedral+monoclinic.	………	………	………	………	………	
	33.3:66.7	800	20	………	………	………	………	………	………	………	………	
		1350	6	……	………	………	………	……	………	………	………	
		1500	6	Cr_2_O_3 ss_+Ga_2_O_3 ss_	Corundum+beta gallia.	Rhombohedral+monoclinic.	………	……	………	……	……	
	20:80	800	20	………	………	……	……	………	……	………	………	
		1350	6	………	………	………	……	……	………	………	………	
		1500	6	Ga_2_O_3 ss_	Beta gallia	Monoclinic	………	……	……	………	……	………
**Cr^+3^ and larger cations**												
La_2_O_3_·Cr_2_O_3_	50:50	……	………	La_2_O_3_·Cr_2_O_3_	Perovskite	Orthorhombic	5.477	5.514	7.755	……	………	Geller [6].
Pr_2_O_3_·Cr_2_O_3_	50:50	……	………	Pr_2_O_3_·Cr_2_O_3_	Perovskite	Orthorhombic	5.444	5.484	7.710	………	……	Geller [6].
Nd_2_O_3_·Cr_2_O_3_	50:50	……	………	Nd_2_O_3_·Cr_2_O_3_	Perovskite	Orthorhombic	5.412	5.494	7.695	………	……	Geller [6].
Sm_2_O_3_·Cr_2_O_3_	66.7:33.3	800	20	………	……	……	………	………	……	………	………	
		1350	6	……	……	……	………	………	………	………	……	
		1500	6	Sm_2_O_3_+Sm_2_O_3_·Cr_2_O_3_	B-type+perovskite	Monoclinic+orthorhombic.	……	………	………	………	……	
	50:50	………	………	Sm_2_O_3_·Cr_2_O_3_	Perovskite	Orthorhombic	5.372	5.502	7.650	………	Results confirmed in present work for specimen heat treated at 1600 °C.	Geller [6].
	37.5:62.5	800	20	………	……	……	………	………	………	………	………	
		1350	6	……	……	……	……	……	……	……	……	
		1500	6	Sm_2_O_3_·Cr_2_O_3_+Cr_2_O_3_	Perovskite+corundum	Orthorhombic+rhomboliedral	………	………	……	………	………	
Eu_2_O_3_–Cr_2_O_3_	50:50	1000	6	…………	………	……	………	……	……	………	……	
		1600	6	Eu_2_O_3_·Cr_2_O_3_	Perovskite	Orthorhombic	5.33	7.61	5.50	……	……	
Gd_2_O_3_–Cr_2_O_3_	66.7:33.3	800	20	……	………	……	……	……	……	……	……	
		1350	6	………	………	………	………	……	………	……	……	
		1500	6	Gd_2_O_3_+Gd_2_O_3_·Cr_2_O_3_	B-type+perovskite	Monoclinic+orthorhombic	………	……	……	………	……	
	50:50	……	………	Gd_2_O_3_·Cr_2_O_3_	Perovskite	Orthorhombic	5.312	5.514	7.611	……	Results confirmed in present work for specimen heat treated at 1600 °C.	Geller [6].
	37 5:62.5	800	20	………	………	………	……	……	……	………	……	
		1350	6	……	………	……	………	……	……	………	………	
		1.500	6	Gd_2_O_3_·Cr_2_O_3_+Cr_2_O_3_	Perovskite+corundum	Orthorhombic+rhombohedral	………	……	……	……	……	
Dy_2_O_3_–C_12_O_3_	50:50	1000	6	…………	…………	………	……	………	………	………	………	
		1600	6	Dy_2_O_3_·Cr_2_O_3_	Perovskite	Orthorhombic	5.26	7.50	5.51	……	………	
Y_2_O_3_–Cr_2_O_3_	50:50			Y_2_O_3_·Cr_2_O_3_	Perovskite	Orthorhombic	5.247	5.518	7.540	……	……	Geller [6].
Er_2_O_3_–Cr_2_O_3_	50:50	1000	6	…………	…………	…………	………	……	……	……	……	
		1600	6	Er_2_O_3_·Cr_2_O_3_	Perovskite	Orthorhombic	5.22	7.51	5.51	……	……	
Yb_2_O_3_–Cr_2_O_3_	66.7:33.3	800	20	………	…………	………	………	………	………	……	………	
		1350	6	…………	………	………	………	……	……	……	………	
		1500	6	Yb_2_O_3_+Yb_2_O_3_·Cr_2_O_3_	C-type+perovskite	Cubic+orthorhombic	……	……	………	………	……	
	50:50	1000	6	………	………	………	……	………	……	……	……	
		1600	6	Yb_2_O_3_·Cr_2_O_3_	Perovskite	Orthorhombic.	5.18	7.51	5.49	………	……	
	37.5:62.5	800	20	……	………	……	………	………	……	………	………	
		1350	6	………	………	……	………	………	………	………	………	
		1500	6	Yb_2_O_3_·Cr_2_O_3_+Cr_2_O_3_	Perovskite+corundum	Orthorhombic+rhombohedral	……	………	……	……	……	
Lu_2_O_3_–Cr_2_O_3_	50:50	1000	6	………	…………	………	……	………	………	……	………	
		1600	6	Lu_2_O_3_·Cr_2_O_3_	Perovskite	Orthorhombic	5.17	7.46	5.49	………	……	
In_2_O_3_–Cr_2_O	50:50	1000	6	………	………	………	………	……	………	………	………	
		1600	46	In_2_O_3 ss_+Cr_2_O_3 ss_	C-type+corundum	Cubic+rhombohedral	……	………	……	……	……	
Sc_2_O_3_–Cr_2_O_3_	80:20	800	20	………	…………	………	……	………	……	……	………	
		1350	6	…………	…………	…………	……	……	………	………	……	
		1500	6	Sc_2_O_3 ss_+(1−*x*)Sc_2_O_3_·*x*Cr_2_O_3 ss_	C-type+unknown	Cubic+unknown	……	……	………	………	……	
	75:25	800	6	……	…………	…………	……	………	…………	……	………	
		1350	6	………	………	………	……	……	………	………	………	
		1500	6	(1−*x*)Sc_2_O_3_·*x*Cr_2_O_3 ss_	Unknown	Unknown	………	………	……	……	Unknown phase similar to kappa alumina type phase.	
	66.7:33.3	800	20	……	………	…………	……	……	……	……	……	
		1600	6	(1—*x*)Sc_2_O_3_·*x*Cr_2_O_3 ss_ +Cr_2_O_3 ss_	Unknown+corundum	Unknown+rhombohedral	………	………	……	……	………	
Sc_2_O_3_–Cr_2_O_3_	50:50	1000	20	………	……	……	………	……	………	………	………	
		1600	6	(1−*x*)Sc_2_O_3_·*x*Cr_2_O_3 ss_+Cr_2_O_3 ss_	Unknown+corundum.	Unknown+rhombohedral.	……	……	………	………	……	
Fe_2_O_3_–Cr_2_O_3_^3f^	50:50	1000	6	……	………	……	………	……	………	………	……	
		1350	6	(1−*x*)Fe_2_O_3_·xCr_2_O_3 ss_	Corundum	Rhombohedral	………	……	………	……	……	
**Fe^+3^ and larger cations**												
La_2_O_3_–Fe_2_O_3_	50:50	1500	1	La_2_O_3_·Fe_2_O_3_	Perovskite	Orthorhombic	5.545	7.851	5.562	………	…………	Roth [4].
Pr_2_O_3_–Fe_2_O_3_	50:50	………	……	Pr_2_O_3_·Fe_2_O_3_	Perovskite	Orthorhombic	5.495	5.578	7.810	………	………	Geller and Wood [5].
Nd_3_O_3_–Fe_2_O_3_	50:50	……	……	Nd_2_O_3_·Fe_2_O_3_	Perovskite	Orthorhombic	5.441	5.573	7.753	……	…………	Geller and Wood [5].
Sm_2_O_3_–Fc_2_O_3_	50:50	……	……	Sm_2_O_3_·Fe_2_O_3_	Perovskite	Orthorhombic	5.394	5.592	7.711	………	………	Geller and Wood [5].
	37.5:62.5	………	……	3Sm_2_O_3_·5Fe_2_O_3_	Garnet	Cubic	12.524	……	………	………	Garnet phase does not form stably in binary mixtures containing Fe_2_O_3_ and trivalent cations larger than Sm^+3^ [34].	Bertaut and Forrat [34].
_2_O_3_–Fe_2_O_3_	50:50	………	……	Eu_2_O_3_·Fe_2_O_3_	Perovskite	Orthorhombic	5.371	5.611	7.686	………	………	Geller and Wood [5].
	37.5:62.5	……	………	3Eu_2_O_3_·5Fe_2_O_3_	Gamet	Cubic	12.518	………	……	………	………	Bertaut and Forrat [34].
Gd_2_O_3_–Fe_2_O_3_^3h^	50:50	………	………	Gd_2_O_3_·Fe_2_O_3_	Perovskite	Orthorhombic	5.346	5.616	7.668	……	Results confirmed in present work for specimen heat treated at 1300 °C.	Geller and Wood [5].
	37.5:62.5	……	……	3Gd_2_O_3_·5Fe_2_O_3_	Garnet	Cubic	12.479	………	……	………	……	Bertaut and Forrat [34].
Dy_2_O_3_–Fe_2_O_3_	66.7:33.3	800	20	………	………	………	………	………	………	………	………	
		1000	20	………	………	………	……	………	………	………	………	
		1350	6	Dy_2_O_3_+Dy_2_O_3_·Fe_2_O_3_	C-type+perovskite	Cubic+orthorhombic	………	……	………	………	………	
	50:50	800	20	……	……	……	………	………	………	………	………	
		1000	65	………	………	……	……	………	………	………	………	
		1300	6	Dy_2_O_3_·Fe_2_O_3_+3Dy_2_O_3_·5Fe_2_O_3_	Perovskite+garnet	Orthorhombic+cubic	……	………	……	………	Nonequilibrium	
		1500	6	Dy_2_O_3_·Fe_2_O_3_	Perovskite	Orthorhombic	5.30	7.62	5.59	……	……	
	37.5:62.5	………	………	3Dy_2_O_3_·5Fe_2_O_3_	Gamet	Cubic	12.414	………	……	………	……	Bertaut and Forrat [34].
Ho_2_O_3_–Fe_2_O_3_	50:50	800	20	………	………	……	………	……	……	………	……	
		1000	65	………	………	………	………	………	………	………	………	
		1350	6	Ho_2_O_3_·Fe_2_O_3_+3Ho_2_O_3_·5Fe_2_O_3_	Perovskite+garnet	Orthorhombic+cubic	………	………	……	………	Nonequilibrium	
		1500	6	Ho_2_O_3_·Fe_2_O_3_	Perovskite	Orthorhombic	5.30	7.58	5.59	………	……	
	37.5:62.5	……	………	3Ho_2_O_3_·5Fe_2_O_3_	Garnet	Cubic	12.380	……	……	………	……	Bertaut and Forrat [34].
Y_2_O_3_–Fe_2_O_3_^3i^	50:50	1500	1	Y_2_O_3_·Fe_2_O_3_	Perovskite	Orthorhombic	5.279	7.609	5.590	………	……	Roth [4].
	37.5:62.5	……	………	3Y_2_O_3_·5Fe_2_O_3_	Garnet	Cubic	12.376	………	………	………	……	Bertaut and Forrat [34].
Er_2_O_3_–Fe_2_O_3_	50:50	800	20	………	……	………	……	………	………	……	………	
		1000	65	………	……	………	………	……	………	………	………	
		1300	6	……	……	………	………	………	………	………	……	
		1500	6	Er_2_O_3_·Fe_2_O_3_	Perovskite	Orthorhombic	5.26	7.58	5.58	………	……	
	37.5:62.5	……	………	3Er_2_O_3_·5Fe_2_O_3_	Garnet	Cubic	12.349	………	……	………	………	Bertaut and Forrat [34].
Tm_2_O_3_–Fe_2_O_3_	37.5:62.5	………	………	3Tm_2_O_3_·5Fe_2_O_3_	Garnet	Cubic	12.325	………	………	………	……	Bertaut and Forrat [34].
Yb_2_O_3_–Fe_2_O_3_	66.7:33.3	800	20	………	………	……	………	………	……	………	………	
		1000	20	……	……	……	………	………	………	………	………	
		1350	6	Yb_2_O_3_+Yb_2_O_3_·Fe_2_O_3_	C-type+perovskite	Cubic+orthorhombic	………	………	………	………	……	
	50:50	800	20	………	………	……	………	……	……	………	………	
		1000	65	……	………	……	………	………	………	………	……	
		1300	6	………	………	……	………	………	………	………	……	
		1500	6	Yb_2_O_3_·Fe_2_O_3_	Perovskite	Orthorhombic	5.22	7.56	5.58	………	………	
	37.5:62.5	………	………	3Yb_2_O_3_·5Fe_2_O_3_	Garnet	Cubic	12.291	……	……	………	……	Bertaut and Forrat [34].
Lu_2_O_3_·Fe_2_O_3_	50:50	800	20	……	………	………	………	………	………	………	………	
		1000	65	………	………	………	……	………	……	……	……	
		1300	6	Lu_2_0_3_·Fe_2_0_3_	Perovskite	Orthorhombic	5.21	7.55	5.55	……	……	
		1500	6	do	do	do	………	………	……	……	……	
	37.5:62.5	………	……	3Lu_2_O_3_·5Fe_2_O_3_	Garnet	Cubic	12.277	……	……	………	……	Bertaut and Forrat [34].
In_2_O_3_–Fe_2_O_3_	50:50	800	20	……	………	…………	………	……	………	………	……	
		1000	65	………	………	……	……	……	………	………	………	
		1350	6	In_2_O_3 ss_+Fe_2_O_3 ss_	C-type+corundum	Cubic+rhombohedral	……	………	………	………	……	
		1500	46	do	do	do	……	………	……	………	……	
Sc_2_O_3_–Fe_2_O_3_^3j^	50:50	800	20	……	……	……	………	……	……	……	………	
		1000	65	………	………	………	………	……	………	………	……	
		1350	6	Sc_2_O_3 ss_+Fe_2_O_3 ss_	C-type+corundum	Cubic+rhombohedral.	……	………	……	………	Nonequilibrium	
		1500	6	Sc_2_O_3 ss_	C-type	Cubic	……	………	……	………	………	
	37.5:62.5	800	20	………	………	……	………	………	………	………	……	
		1000	20	……	………	……	………	……	………	………	……	
		1350	6	Sc_2_O_3 ss_+Fe_2_O_3 ss_	C-type+corundum	Cubic+rhombohedral.	……	……	………	……	……	
**Sc^+3^ and larger cations**												
La_2_O_3_–Sc_2_O_3_	50:50	………	………	La_2_O_3_·Sc_2_O_3_	Perovskite	Orthorhombic	5.678	5.787	8.098	………	……	Geller [6].
Pr_2_O_3_–Sc_2_O_3_	50:50	………	………	Pr_2_O_3_·Sc_2_O_3_	Perovskite	Orthorhombic	5.615	5.776	8.027	……	………	Geller [6].
Nd_2_O_3_–Sc_2_O_3_	50:50	……	………	Nd_2_O_3_·Sc_2_O_3_	Perovskite	Orthorhombic	5.574	5.771	7.998	……	………	Geller [6].
Sm_2_O_3_–Sc_2_O_3_	50:50	1350	6	……	………	………	……	………	………	………	………	
		1650	9.5	Sm_2_O_3_·Sc_2_O_3_	Perovskite	Orthorhombic	5.53	7.95	5.76	………	………	
Eu_2_O_3_–Sc_2_O_3_	50:50	1350	6	……	………	………	………	……	……	………	……	
		1650	9.5	Eu_2_O_3_·Sc_2_O_3_	Perovskite	Orthorhombic	5.51	7.94	5.76	………	……	
Gd_2_O_3_–Sc_2_O_3_	66.7:33.3	1350	6	………	………	……	………	……	………	………	……	
		1500	6	………	……	………	……	……	………	………	……	
		1650	6	Gd_2_O_3 ss_+Gd_2_O_3_·Sc_2_O_3_	B-type+perovskite	Monoclinic+orthorhombic	………	………	………	………	……	
	50:50	……	……	Gd_2_O_3_·Sc_2_O_3_	Perovskite	Orthorhombic	5.487	5.756	7.925	……	Results confirmed in present work for specimen heat treated at 1650 °C.	Geller [6].
	37.5:62.5	1350	6	………	………	……	………	………	………	………	………	
		1500	6	Gd_2_O_3_·Sc_2_O_3_+Sc_2_O_3 ss_	Perovskite+C-type	Orthorhombic+cubic	……	……	………	………	……	
		1650	6	do	do	do	………	………	………	………	………	
Dy_2_O_3_–Sc_2_O_3_	66.7:33.3	1350	6	………	………	……	……	………	………	……	………	
		1500	6	Dy_2_O_3 ss_+Dy_2_O_3_·Sc_2_O_3_	C-type+perovskite	Cubic-orthorhombic.	………	……	………	……	……	
	50:50	1350	6	……	……	……	………	………	………	………	………	
		1650	9.5	Dy_2_O_3_·Sc_2_O_3_	Perovskite	Orthorhombic	5.43	7.89	5.71	………	………	
Ho_2_O_3_–Sc_2_O_3_	50:50	1350	6	………	………	………	………	……	……	………	………	
		1650	9.5	……	………	………	……	……	………	……	……	
		1875	0.3	Ho_2_O_3 ss_+Ho_2_O_3_·Sc_2_O_3_+Sc_2_O_3 ss_	C-type+perovskite+C-type	Cubic+orthorhombic+cubic	5.42	7.87	5.71	………	Nonequilibrium listed dimensions for perovskite phase in mixture.	
		1900	1	do	do	do	………	………	………	………	Nonequilibrium	
		1950	1	do	do	do	………	………	………	……	do	
		2000	0.3	(1−*x*)Ho_2_O_3_·*x*Sc_2_O_3 ss_ +Ho_2_O_3_·Sc_2_O_3_	C-type+perovskite	Cubic+orthorhombic	………	…………	………	……	Nonequilibrium; amount of perovskite phase small. Equilibrium probably single phase C-type solid solution.	
Y_2_O_3_–Sc_2_O_3_	50:50	1350	6	……	……	………	………	……	………	……	………	
		1650	9.5	Y_2_O_3 ss_+Y_2_O_3_·Sc_2_O_3_+Sc_2_O_3 ss_	C-type+perovskite+C-type	Cubic+orthorhombic+cubic	………	………	………	………	Nonequilibrium	
		1890	0.3	(1−*x*)Y_2_O_3_·*x*Sc_2_O_3 ss_	C-type	Cubic	………	……	………	………	……	
Er_2_O_3_–Sc_2_O_3_	50:50	1350	6	……	……	………	………	………	………	………	………	
		1650	9.5	Er_2_O_3 ss_+Sc_2_O_3 ss_	C-type+C-type	Cubic+cubic	………	………	………	……	Nonequilibrium	
		1850	0.7	(1−*x*)Er_2_O_3_·Sc_2_O_3 ss_	C-type	Cubic	………	………	………	………	……	
Yb_2_O_3_–Sc_2_O_3_	50:50	1350	6	……	………	………	………	………	………	………	……	
		1650	9.5	Yb_2_O_3 ss_+Sc_2_O_3 ss_	C-type+C-type	Cubic+cubic	………	………	………	………	Nonequilibrium	
		1850	0.3	(1−*x*)Yb_2_O_3_·*x*Sc_2_O_3 ss_	C-type	Cubic	……	………	……	………	……	
Lu_2_O_3_–Sc_2_O_3_	50:50	1350	6	………	………	………	………	………	………	………	………	
		1650	9.5	(1−*x*)Lu_2_O_3_·*x*Sc_2_O_3 ss_	C-type	Cubic	………	………	………	………	……	
In_2_O_3_–Sc_2_O_3_	50:50	800	20	………	………	………	………	………	………	………	………	
		1350	46	(1−*x*)In_2_O_3_·*x*Sc_2_O_3 ss_	C-type	Cubic	……	………	………	……	………	
**In^+3^ and larger cations**												
La_2_O_3_–In_2_O_3_	50:50	1350	0.5	La_2_O_3_·In_2_O_3_	Perovskite	Orthorhombic	5.723	8.207	5.914	………	………	Roth [4].
Nd_2_O_3_–In_2_O_3_	95:5	800	20	……	………	………	………	………	………	………	………	
		1350	6	……	……	………	………	………	………	………	……	
		1500	42	(1−*x*)Nd_2_O_3_·*x*In_2_O_3 ss_	B-type	Monoelinic	………	………	………	………	……	
	85:15	800	20	……	……	……	………	……	………	………	……	
		1350	6	……	……	……	………	………	………	………	………	
		1500	42	(1−*x*)Nd_2_O_3_·*x*In_2_O_3 ss_+Nd_2_O_3_·In_2_O_3_	B-type+perovskite	Monoclinic+orthorhombic.	……	……	……	………	……	
	75:25	800	20	……	………	……	………	……	……	……	………	
		1350	6	……	………	……	……	………	………	………	………	
		1500	42	(1−*x*)Nd_2_O_3_·*x*In_2_O_3 ss_ +Nd_2_O_3_In_2_O_3_	B-type+perovskite	Monoclinic+orthorhombic.	………	………	………	………	……	
	66.7:33.3	800	20	………	…………	………	………	………	………	……	………	
		1350	6				……	……	……	……	……	
		1500	4 2	(1−*x*)Nd_2_O_3_·*x*In_2_O_3 ss_+Nd_2_O_3_·In_2_O_3_	B-type+perovskite	Monoclinic+orthorhombic.	……	……		……	……	
	50:50	1350	0.5	Nd_2_O_3_·In_2_O_3_	Perovskite	Orthorhombic	5.627	8.121	5.891	……	……	Roth [4]
	33.3:66.7	800	20	……	……	……	……	……	……	……	……	
		1350	6	……	……	……	……	……	……	……	……	
		1500	4 2	Nd_2_O_3_·In_2_O_3_+In_2_O_3_	Perovskite+C-type	Orthorhombic+cubic	……	……	……	……	……	
Sm_2_O_3_–In_2_O_3_	66.7:33.3	800	20	……	……	……	……	……	……	……	……	
		1350	6	……	……	……	……	……	……	……	……	
		1500	4 2	Sm_2_O_3 ss_+Sm_2_O_3_·In_2_O_3_	B-type+perovskite	Monoclinic+orthorhombic	……	……	……	……	……	
	50:50	1350	0.5	Sm_2_O_3_·In_2_O_3_	Perovskite	Orthorhombic	5.589	8.082	5.886	……	……	Roth [4]
	33.3:66.7	800	20	……	……	……	……	……	……	……	……	
		1350	6	……	……	……	……	……	……	……	……	
		1500	4 2	Sm_2_O_3_·In_2_O_3_+In_2_O_3 ss_	Perovskite+C-type	Orthorhombic+cubic	……	……	……	……	……	
Eu_2_O_3_–In_2_O_3_^3k^	50:50	800	20	……	……	……	……	……	……	……	……	
		1350	6	……	……	……	……	……	……	……	……	
		1650	4 2	Eu_2_O_3_·In_2_O_3_	Unknown	Unknown	……	……	……	……	Pseudo hexagonal symmetry	Schneider [23]
Gd_2_O_3_–In_2_O_3_	66.7:33.3	800	20	……	……	……	……	……	……	……	……	
		1350	6	……	……	……	……	……	……	……	……	
		1600	4 2	Gd_2_O_3 ss_+Gd_2_O_3_·In_2_O_3_	B-type+unknown	Monoclinic+unknown.	……	……	……	……	……	
	50:50	800	20	……	……	……	……	……	……	……	……	
		1350	6	……	……	……	……	……	……	……	……	
		1600	4 1	Gd_2_O_3_·In_2_O_3_	Unknown	Unknown	……	……	……	……	Isostructural with Eu_2_O_3_·In_2_O_3_	Schneider [23]
		1650	4 2	do	do	do	……	……	……	……	……	
	33.3:66.7	800	20	……	……	……	……	……	……	……	……	
		1350	6	……	……	……	……	……	……	……	……	
		1600	4 2	Gd_2_O_3_·In_2_O_3 ss_+In_2_O_2 ss_	Unknown+C-type	Unknown+cubic	……	……	……	……	……	
Dy_2_O_3_–In_2_O_3_	95:5	800	20	……	……	……	……	……	……	……	……	
		1350	6	……	……	……	……	……	……	……	……	
		1600	4 2	Dy_2_O_3 ss_+(1*−x*) Dy_2_O_3_·*x*In_2_O_3 ss_	C-type+B-type	Cubic+monoclinic	……	……	……	……	……	
	85:5	800	20	……	……	……	……	……	……	……	……	
		1350	6	……	……	……	……	……	……	……	……	
		1600	4 2	Dy_2_O_3 ss_+(1*−x*) Dy_2_O_3_·*x*In_2_O_3 ss_	C-type+B-type	Cubic+monoclinic	……	……	……	……	……	
	75:25	800	20	……	……	……	……	……	……	……	……	
		1350	6	……	……	……	……	……	……	……	……	
		1600	4 2	(1*−x*)Dy_2_O_3_·*x*In_2_O_3 ss_	B-type	Monoclinic	……	……	……	……	……	
	66.7:33.3	800	20	……	……	……	……	……	……	……	……	
		1350	6	……	……	……	……	……	……	……	……	
		1600	4 2	(1*−x*)Dy_2_O_3_·*x*In_2_O_3 ss_ + Dy_2_O_3_·In_2_O_3 ss_	B-type+unknown	Monoclinic+unknown	……	……	……	……	……	
	50:50	800	20	……	……	……	……	……	……	……	……	
		1350	6	……	……	……	……	……	……	……	……	
		1600	4 1	Dy_2_O_3_·In_2_O_3_	Unknown	Unknown	……	……	……	……	Isostructural with Eu_2_O_3_·In_2_O_3_ [23]	Schneider [23]
		1650	4 3	(1*−x*)Dy_2_O_3_·*x*In_2_O_3 ss_+In_2_O_3 ss_	B-type+C-type	Monoclinic+cubic	……	……	……	……	……	
	33.3:66.7	800	20	……	……	……	……	……	……	……	……	
		1350	6	……	……	……	……	……	……	……	……	
		1600	4 2	Dy_2_O_3_·In_2_O_3 ss_+In_2_O_3 ss_	Unknown+C-type	Unknown+cubic	……	……	……	……	……	
Ho_2_O_3_–In_2_O_3_	50:50	800	20	……	……	……	……	……	……	……	……	
		1350	6	……	……	……	……	……	……	……	……	
		1600	4 1	(1−*x*)Ho_2_O_3_·*x*In_2_O_3 ss_	C-type	Cubic	……	……	……	……	……	
Er_2_O_3_–In_2_O_3_	50:50	800	20	……	……	……	……	……	……	……	……	
		1350	6	……	……	……	……	……	……	……	……	
		1600	4 1	(1−*x*)Er_2_O_3_·*x*In_2_O_3 ss_	C-type	Cubic	……	……	……	……	……	
Tm_2_O_3_–In_2_O_3_	50:50	800	20	……	……	……	……	……	……	……	……	
		1350	6	……	……	……	……	……	……	……	……	
		1600	4 1	(1−*x*)Tm_2_O_3_·*x*In_2_O_3 ss_	C-type	Cubic	……	……	……	……	……	
Yb_2_O_3_–In_2_O_3_	50:50	800	20	……	……	……	……	……	……	……	……	
		1350	6	……	……	……	……	……	……	……	……	
		1600	4 1	(1−*x*)Yb_2_O_3_·*x*In_2_O_3 ss_	C-type	Cubic	……	……	……	……	……	
Lu_2_O_3_–In_2_O_3_	50:50	800	20	……	……	……	……	……	……	……	……	
		1350	6	……	……	……	……	……	……	……	……	
		1600	4 1	(1−*x*)Lu_2_O_3_·*x*In_2_O_3 ss_	C-type	Cubic	……	……	……	……	……	

1 All specimens in present work slow cooled except where noted. Each heat treatment includes all previously listed lower temperature heatings given for the same composition.

2 When no references are given, data was obtained in present investigation.

3 The phase diagram for this system has been reported: (a) [22], (b) [10], (c) [26], (d) [27], (e) [28], (f) [10], (g) [29], (h) [22], (i) [18], (j) [30], (k) [23].

4 Specimen heated in sealed Pt tubes and quenched.

**Table 2:** X-ray powder diffraction data for (1−x) *Fe*_2_*O*_3_ · x*Al*_2_*O*_3 ss_ (53Fe_2_O_3_ : 47Al_2_O_3_ mixture)

*hkl*^1^	*d^2^*	*I*^3^	$\frac{1}{d^{2}}$	
			obs	cal
				
	*A*		*A*^−2^	*A*^−2^
100	6.03	25	0.0253	0.0253
020	4.64	23	.0464	.0470
111	3.90	18	.0657	.0656
121	} 3.144	38	.1012	$\{\begin{array}{c} .1008\\ .1012 \end{array}$
220				
130	2.899	38	.1190	.1193
221	2.658	100	.1415	.1415
131	} 2.497	29	.1604	$\{\begin{array}{c} .1596\\ .1610 \end{array}$
002				
012	2.407	18	.1726	.1728
102	} 2.393	34	.1747	$\{\begin{array}{c} .1746\\ .1741 \end{array}$
311				
040	2.306	14	.1881	.1881
022	2.193	23	.2080	.2080
321	2.186	38	.2093	.2093
400	2.146	13	.2171	.2171
122	2.125	21	.2214	.2216
331	1.9000	29	.2679	.2681
042	1.6920	20	.3493	.3491
123	1.5377	16	.4226	.4229

1 Based on orthorhombic cell with *a*=8.59 A, *b*=9.23 A, and *c*=4.98 A.

2 Interplanar spacing.

3 Relative intensity.

**Table 3:** X-ray powder diffraction data for (1−x)*In*_2_*O*_3_·x*Ga*_2_*O*_3__ss_, (1−x)*Sc*_2_*O*_3_·x*Ga*_2_*O*_3__ss_, and (1−x)*Sc*_2_*O*_3_·x*Cr*_2_*O*_3__ss_

50In_2_O_3_:50Ga_2_O_3_ mixture		37.5Sc_2_O_3_:62.5Ga_2_O_3_ mixture		75Sc_2_O_3_:25Cr_2_O_3_ mixture	
		
*d*^1^	*I^2^*	*d*^1^	*I*^2^	*d*^1^	*I*^2^
					
*A*		*A*		*A*	
9.72	13	4.73	12	4.44	18
6.83	18	3.404	36	3.559	21
4.88	9	3.110	39	3.400	59
4.84	17	3.034	8	3.046	57
3.423	11	2.889	29	2.763	100
3.183	37	2.878	100	2.687	45
2.279	57	2.852	100	2.653	12
2.917	100	2.653	88	2.531	15
2.851	11	2.598	16	2.501	52
2.710	100	2.486	11	2.417	9
2.661	57	2.385	88	1.9713	13
2.590	9	2.365	90	1.9465	49
2.437	80	2.247	13	1.9072	80
2.426	80	2.231	8	1.7984	11
2.363	11	2.144	14	1.7172	16
2.298	15	2.131	24	1.6573	49
2.279	8	1.830	6	1.6247	15
2.177	13	1.7905	52	1.6861	27
2.031	13	1.7836	39	1.4903	11
1.9434	8	1.7077	46	1.4666	15
1.9155	9	1.6800	100	1.4395	10
1.8273	33	1.6017	52	1.4221	10
1.8200	34	1.5858	10	1.4127	10
1.7190	100	1.5793	28	1.3902	10
1.6709	8			1.3824	10
1.6345	25	1.5167	24	1.3442	10
1.6148	23			1.3180	11
1.6055	8	1.4933	10	1.2514	11
1.5494	31	1.4890	10		
1.5253	11	1.4839	49		
		1.4447	54		
1.5217	11				
1.5176	24	1.4328	11		
1.5158	32				
1.4784	26	1.4281	24		
1.4756	39	1.4158	42		
		1.3966	48		
1.4592	14	1.3640	9		
1.4551	15				
1.4491	30	1.3496	11		
1.4265	42				
1.3937	14	1.3466	11		
		1.3267	13		
1.3816	9	1.3048	18		
1.3558	14				
1.3310	8				

1 Interplanar spacing.

2 Relative intensity.

**Table 4:** X-ray powder diffraction data for (1−x)*Sc*_2_*O*_3_·x*Al*_2_*O*_3 ss_ (50Sc_2_O_3_: 50A*l*_2_O_3_ mixture)

Rhom. *hkl*^1^	*d*^2^	*I*^3^	$\frac{1}{d^{2}}\text{obs}$	$\frac{1}{d^{2}}\text{cal}$
				
	*A*		*A*^−2^	*A*^−2^
222	2.842	29	0.1238	0.1238
$22\bar{2}$	2.687	100	.1385	.1385
040	2.359	21	.1797	.1798
$04\bar{1}/23\bar{2}$	2.265	5	.1940	.1948
$22\bar{3}$	2.241	5	.1991	.1984
240	2.138	7	.2185	.2174
332	2.004	9	.2491	.2500
$04\bar{3}$	1.8496	5	.2923	.2920
$15\bar{2}$	1.7051	21	.3440	.3436
$04\bar{4}$	1.6350	28	.3741	.3741
$26\bar{2}$	1.4180	12	.4973	.4979
$\bar{2}6\bar{2}$	1.3990	5	.5123	.5130

1 Rhombohedral cell, *a*=9.45 A, *α*=87.4°

Hexagonal cell, *a*=13.07 A, *c*=17.05 A.

2 Interplanar spacing.

3 Relative intensity.

**Table 5:** X-ray powder diffraction data for 3 *Gd*_2_*O*_3_·*Ga*_2_*O*

*d*^1^	*I*^2^	*d*^1^	*I*^2^
			
4.53	20	2.005	30
4.11	13	1.9918	15
3.204	14		
3.054	100	1.9027	15
3.025	57	1.8349	32
		1.8097	14
2.990	27	1.7672	17
2.908	22	1.7184	17
2.824	29		
2.630	17	1.6808	17
2.301	15	1.6450	24
		1.5788	24
2.241	17	1.5456	17
2.199	13	1.5276	25
2.032	39		

1 Interplanar spacing.

2 Relative intensity.

**Table 6:** X-ray powder diffraction data for 2Y_2_O_3_·Al_2_O_3_

*hkl*	Warshaw and Roy [22]		Present work		*hkl*^1^	Warshaw and Roy [22]		Present work	
	*d*^2^	*I*^3^	*d*^2^	*I*^3^		*d*^2^	*I*^3^	*d*^2^	*I*^3^
									
110	7.46	10	7.41	63				1.9811	16
200	5.28	3	5.26	16				1.9449	15
210	4.71	22	4.69	100				1.9163	12
			4.54	16				1.9027	16
220	3.71	7	3.705	19	440	1.843	18	1.8426	80
310	3.33	33	3.326	100	522/441^1^	1.828	20	1.8298	85
301	3.01	100	3.011	100+	522/441^4^	1.816	19	1.8164	81
320	2.91	94	2.908	100	530/433	1.793	7	1.7921	32
			2.884	47	600/442	1.732	10	1.7317	32
400	2.62	17	2.615	48	610^4^	1.722	13	1.7235	61
410/322^4^	2.56	10	2.559	64	610^4^	1.716	13		
410/322^4^	2.53	10	2.538	29	611/532	1.711	8		
			2.523	61				1.6279	27
			2.486	21				1.6236	20
			2.470	39				1.6126	23
330/411	2.46	9	2.454	37	622	1.575	9	1.5759	41
421^4^	2.29	7	2.291	43				1.5661	45
421^4^	2.27	7	2.274	28	630/542	1.561	12	1.5621	60
			2.129	12	631	1.551	7	1.5504	44
500/430	2.07	22	2.090	13				1.5065	32
510/431	2.06	12	2.063	87	543/710/550	1.484	4	1.4847	27
			2.046	41				1.4809	24
								1.4541	15
					720/641	1.436	4	1.4379	23
								1.3850	15

1 Based on cubic cell with *a*=10.40 A [22].

2 Interplanar spacing.

3 Relative intensity.

4 “Splitting may represent a possible rhombohedral distortion of the cubic lattice” [22].

## Footnotes

1 Figures in brackets indicatet he literature references at the end of this paper.

2 Radii values are according to Ahrens [8] with the exception of Y^+3^, In^+3^ and Sc^+3^ which were taken from Roth and Schneider [9]. The following radii values were used throughout this report: La^+3^–1.14 A, Ce^+3^–1.07 A, Pr^+3^–1.06 A, Nd^+3^–1.04 A, Sm^+3^–1.00 A, Eu^+3^–.98 A, Gd^+3^–.97 A, Tb^+3^–.93 A, Dy^+3^–.92 A, Ho^+3^–.91 A, Y^+3^–~.91 A, Er^+3^–.89 A, Tm^+3^–.87 A, Yb^+3^–.86 A, Lu^+3^–85 A, In^+3^–.77 A, Sc^+3^–.68 A, Fe^+3^–64 A, Cr^+3^–63 A, Ga^+3^–.62 A, and Al^+3^–.51 A.

3 The symbol “Ln” represents the lanthanide series, lanthanum through lutecium.

4 It has been suggested by S. Geller in a private communication that the solid solution may be of the interstitual and/or vacancy types instead of substitutional and thus result in a defect structure.

5 Unit cell dimensions converted from *kX* units.
